# Elite donors and stable marker–trait associations for grain iron and zinc biofortification in rice under organic and inorganic production systems

**DOI:** 10.3389/fpls.2026.1822204

**Published:** 2026-07-10

**Authors:** Sushmitharaj Dhanalakshmi Veeraraj, Karthika Rajendran

**Affiliations:** VIT School of Agricultural Innovations and Advanced Learning (VAIAL), Vellore Institute of Technology, Vellore, Tamil Nadu, India

**Keywords:** biofortification, grain micronutrients, marker-trait associations, organic agriculture, rice landraces, SSR markers

## Abstract

**Background:**

Micronutrient malnutrition caused by iron (Fe) and zinc (Zn) deficiency remains a major concern for rice-based diets. Genetic enhancement for higher grain Fe and Zn concentration, along with grain yield, is an important breeding objective under organic and inorganic production systems. To develop genetic resources for sustainable biofortification, this study evaluated phenotypic variation, genetic diversity, and marker–trait associations (MTAs) for grain Fe and Zn concentration in a diverse set of 80 rice genotypes, which included both landraces and modern varieties.

**Results:**

Substantial genotypic variation and genotype × environment interactions were observed for both micronutrients, with grain Fe showing greater environmental responsiveness than Zn. Moderate genetic variability coupled with high heritability indicated good scope for selection. Coloured rice landraces consistently recorded higher grain Fe and Zn concentration compared to white rice genotypes. BLUP-based ranking combined with mean performance across organic and inorganic systems identified Kamban samba, Garudan samba, Basmati, Annam alagi, Valan samba, and Sempalai, as superior donors for grain micronutrient improvement. Notably, correlation analysis revealed weak and non-significant associations between grain micronutrients and key agronomic traits, including grain yield, suggesting that nutritional enhancement can be achieved without compromising productivity. Molecular analysis using 32 previously reported Fe and Zn-related SSR markers together with five gene-specific markers revealed moderate allelic diversity with weak population stratification. Single-marker analysis (SMA) identified 15 significant MTAs, including both stable and production system-specific associations. Gene-specific markers such as OsYSL2b and OsNRAMP7 showed significant associations, particularly under organic production systems.

**Conclusion:**

Grain colour and production systems significantly influenced micronutrient accumulation. The weak association between grain micronutrients and agronomic traits indicates strong potential for developing high-yielding and nutrient-dense rice genotypes without adverse linkage drag. The identification of elite donor genotypes and associated molecular markers provides a useful framework for marker-assisted biofortification breeding under diverse and sustainable production systems.

## 1 Introduction

Worldwide, more than 2 billion people in developing countries are affected by iron (Fe) and zinc (Zn) micronutrient deficiencies, commonly termed as “hidden hunger” (Bouis and Saltzman, 2017). Fe and Zn are considered as essential micronutrients (Kaur et al., 2025), Fe is essential for the synthesis of haemoglobin and myoglobin, functions as a cofactor in enzymes involved in energy metabolism and immune responses (Stoltzfus et al., 2004). Zn is required for gene expression, cell division, growth, reproduction, and immune function (Brown et al., 2001; Brown, 2004).

Rice (*Oryza sativa* L.) is the primary staple food for a large proportion of global population but contains only minimal quantities of these micronutrients (Saleh et al., 2019). Moreover, the widespread practice of consuming polished rice further reduces grain Fe and Zn concentration, thereby aggravating “hidden hunger”, particularly amongst Asian population. Fe deficiency is linked to anaemia, higher maternal mortality, early births, low birth weight, and problems with cognitive and motor development (Bouis, 2003). Likewise, Zn deficiency is linked to stunted growth, compromised immune function, skeletal abnormalities, and increased risk of childhood mortality (Roohani et al., 2013; Allai et al., 2021; Stevens et al., 2022). Globally, malnutrition is a serious public health concern and is a major cause of childhood deaths (UNICEF, 2023), with almost two billion people suffering from micronutrient deficiency (Bouis and Welch, 2010).

To address this nutritional gap, since 2003, HarvestPlus and its partner institutions has given considerable attention towards plant breeding based biofortification programs directed towards enhancing Fe and Zn concentration of grains (Bouis and Saltzman, 2017). Earlier studies have reported wide genetic variability for these micronutrients in natural rice germplasm, indicating the availability of useful donor sources for micronutrient improvement (Roy and Sharma, 2014; Raza et al., 2019b; Pradhan et al., 2020; Anusha et al., 2021). Plant breeding-based biofortification is recognised as the one of the most economical and sustainable approaches to enhance grain micronutrient concentration. The presence of genetic variability for grain Fe and Zn in rice germplasm therefore forms a strong basis for biofortification efforts. In this context, the HarvestPlus initiative under the Consultative Group on International Agricultural Research (CGIAR) has defined breeding targets of 13 mg kg^-^¹ Fe and 28 mg kg^-^¹ Zn in polished biofortified rice, which are expected to meet approximately 30% of the estimated average requirement (EAR) for daily micronutrient intake (Senguttuvel et al., 2023). The recent release of Zn rich variety Spoorthi (GNV 1906) in India highlights the feasibility of this approach.

Additionally, high-throughput equipment Energy-dispersive X-ray fluorescence (ED-XRF) has recently been preferred for Fe and Zn estimation. The efficiency of ED-XRF is 200–300 samples per day, and it is a non-destructive technique suitable for large-scale analysis. It offers a rapid and relatively accurate means of quantification compared to traditional destructive methods like atomic absorption spectrometry (AAS) and inductively coupled plasma optical emission spectrometry (ICP-OES) (Paltridge et al., 2012a; Nachimuthu et al., 2014; Chandu et al., 2020). However, it is important to note that ED-XRF may have lower analytical precision and sensitivity compared to ICP-OES, particularly at low micronutrient concentrations, and therefore requires careful calibration and validation (Paltridge et al., 2012b).

A clear understanding of genetic relationships between genotypes is, therefore, a prerequisite for successful rice biofortification breeding programs (Roy et al., 2015; Sanghamitra et al., 2022). The genetic potential available in rice germplasm for grain Fe and Zn concentration can be efficiently tapped using marker-assisted breeding (MAS) for the development of micronutrient rich rice varieties (Vanlalsanga et al., 2019; Raza et al., 2020; Pradhan and Meena, 2022). Simple sequence repeat (SSR) or microsatellite markers are widely used for genetic diversity analysis owing to their co-dominant nature, high polymorphism, and cost-effectiveness (Li et al., 2017). Although several studies have been conducted on genetic diversity analysis in rice using random (genome wide) and trait-linked SSR markers (Pradhan et al., 2020; Sanghamitra et al., 2022; Kumari et al., 2025). Till date, very limited information is available on diversity analysis using gene-specific SSR markers linked to grain nutrient accumulation.

Besides genetic components, agronomic practices have a significant effect on the accumulation of micronutrients in rice grains (Kumar et al., 2020; Dwiningsih and Alkahtani, 2022). As consumers increasingly demand safe and chemical-free food, organic rice farming has received considerable attention (Babajani et al., 2023; Baird, 2024). Rice landraces, developed through centuries of farmer-led selection, are known for their wider adaptability, resilience, and superior grain nutritional quality, particularly amongst coloured or pigmented rice varieties (Kumar et al., 2025). Organic production systems are found to be well-suited to such landraces. However, despite the success of improvement of certain landraces by pure-line selection ASD16 from kona kuruvai and ASD9 from Avasara samba, the identification of genotypes with stable yield and increased micronutrient concentration under organic systems is limited. Furthermore, the interaction between genotype and management system remains under-explored.

Therefore, exploiting rice landraces for organic agriculture and biofortification is essential to achieve sustainable yield and improved nutritional quality. Understanding the relationship between production systems, grain colour, and micronutrient concentration will facilitate effective utilisation of rice landraces in breeding programmes. In this context, the present study was undertaken to evaluate grain Fe and Zn variability in a diverse panel of rice genotypes comprising landraces and modern varieties grown under organic and inorganic production systems. The study aims to assess the influence of grain colour and production system on micronutrient accumulation, analyse associations between micronutrient concentration and key agronomic traits to identify potential trade-offs, and characterise genetic diversity using both trait- linked and gene-specific SSR markers. These findings will facilitate the identification of stable, nutritionally superior genotypes for future breeding programs.

## 2 Materials and methods

### 2.1 Field evaluation and plant materials

Rice landraces and modern varieties were evaluated for grain micronutrient under organic and inorganic production systems. A total of 80 genotypes, consisting of 62 landraces (30 coloured, 32 white) and 18 modern varieties (1-coloured, 17 white), originating from Tamil Nadu were used in this study (**Supplementary Table 1**). Seeds of the germplasm were obtained from Tamil Nadu Agricultural University, Coimbatore, Agriculture College and Research Institute, Madurai, Krishi Vigyan Kendra, Tirur; and from progressive traditional rice-growing farmers.

All the genotypes were evaluated under both organic and inorganic field conditions at the agriculture farm (12°58’7” N - 79°9’40” E) of VIT School of Agricultural Innovations and Advanced Learning (VAIAL), Vellore Institute of Technology (VIT), Vellore and at farmer’s field (12°57’54” N- 79°10’40” E). The field experiment was conducted under two contrasting production systems, namely organic (low-input) and inorganic (conventional or high-input) conditions. Detailed information on the experimental site, including weather conditions and soil physico-chemical properties under organic and inorganic production systems during the study period, is provided in **Supplementary Table 2**. The experiment was laid out in alpha lattice design with three replications over two consecutive cropping seasons (2023 and 2024). Each genotype was planted in three rows of 3 m length with an inter-row spacing of 20 cm and intra-row spacing of 20 cm. Twenty-five days old seedlings were transplanted into main field under both production systems and standard crop production guidelines specific to organic and inorganic systems were followed (Tamil Nadu Agricultural University (TNAU), 2020). For organic field, a basal application of enriched farmyard manure (FYM) at 750 kg ha^-1^ was applied, followed by top dressing with vermicompost at 1 t ha^-1^ at 25 and 45 days after transplanting and foliar spraying of 3% panchagavya at 15-days intervals until the panicle initiation stage (Tamil Nadu Agricultural University (TNAU), 2026). For the inorganic field, a blanket fertilizer recommendation of 150: 50: 50 kg ha^-1^ (N: P: K) was applied in split doses at basal, tillering and panicle initiation stages. Plants were regularly monitored for visual Fe and Zn deficiency symptoms such as leaf chlorosis and stunted growth during the cropping period; however, no such symptoms were observed under either production systems.

In addition to grain Fe and Zn, selected agronomic traits, including days to fifty percent flowering (DFF), plant height (PH), panicle length (PL), number of productive tillers per plant (NPTP), hundred seed weight (HSW), number of filled grains per panicle (NFGPP) and single plant yield (SPY) were recorded to assess their association with micronutrient concentration and to identify potential trade-offs relevant to biofortification. At physiological maturity, seeds were harvested, threshed, and sun dried to approximately 14% moisture content, and single plant yield (SPY) was recorded at this moisture level. The dried grain samples were stored under cold storage conditions until further analysis for Fe and Zn estimation.

### 2.2 Estimation of grain Fe and Zn

Around 20 g of dried rice grains from each genotype were de-husked using a laboratory rice dehusker (Satake Corporation, Japan). The obtained brown rice samples were carefully cleaned using soft tissue paper to remove adhering dust and surface contaminants (Chandu et al., 2020). To minimise potential micronutrient contamination during processing, the dehusker was thoroughly cleaned between samples and only non-metallic contact surfaces were used during handling.

Grain Fe and Zn concentration were estimated using 5 g of unpolished rice (brown rice) samples following the procedure described by (Rao et al., 2014). Micronutrient analysis was carried out using Energy Dispersive X-Ray Fluorescence Spectrometry (ED-XRF, Model- X- Supreme 8000 Benchtop, Oxford Instruments, UK). The instrument was calibrated using standard reference material prior to analysis and Fe and Zn concentrations were expressed in mg kg^-1^ (ppm).

### 2.3 Molecular characterisation

Total genomic DNA was isolated for all the genotypes from 21-days-old fresh leaf tissues collected during the 2024 cropping season using a modified CTAB (Cetyl tri-methyl ammonium bromide) method (Doyle, 1991). DNA quality and quantity were assessed using a Nanodrop (Thermo Scientific, Wilmington, DE, USA) spectrophotometer (A260/280 ratio) and 0.8% agarose gel electrophoresis with ethidium bromide. DNA was diluted with molecular grade water before performing PCR.

Genotyping was carried out using 32 SSR markers selected from previous studies on grain Fe and Zn concentration in rice based on their reported polymorphism and association with micronutrient traits (Anuradha et al., 2012; Brar et al., 2014; Kiranmayi et al., 2014; Lu et al., 2008; Roja, 2011; Swamy et al., 2011; Swamy et al., 2018; Namdev, 2020; Raza et al., 2020; Tannidi, 2019; Walia, 2015; Anadhu et al., 2025), along with five gene-specific markers targeting functional polymorphisms associated with grain micronutrient accumulation. Detailed information on marker and primer sequences is provided in **Supplementary Table 3**. These markers were utilised for the assessment of molecular diversity and single marker analysis (SMA) to identify marker-trait associations among the rice genotypes.

Series of PCR amplification was performed in C1000 thermocycler (Biorad, USA) using a10µl reaction mixture containing 1 µl genomic DNA (25 ng/µl), 1 µl SSR primer (2 mM), and 8 µl of commercial PCR master mix (Takara Bio Inc., Japan). PCR conditions consisted of initial denaturation at 94 °C for 5 min, followed by 35 cycles of denaturation at 94 °C for 30s, annealing at 55-57 °C for 30s, extension at 72 °C for 1min, and final extension at 72 °C for 10 min. Amplified products were resolved on 3% agarose gel electrophoresis and documented using the gel documentation system. Allele sizes were determined using a 100 bp DNA ladder (Takara Bio Inc., Japan). Clear and reproducible alleles were scored and converted into binary matrix for further analysis.

### 2.4 Statistical analysis

Data on grain micronutrient concentration and key agronomic traits were subjected to mixed model analysis using lme4 package in RStudio (version 2026.01.0 + 392) (Bates et al., 2015). Genotypes were treated as random effects, whilst environments (organic and inorganic), seasons and their interactions were considered fixed effects. The analysis was performed separately for each season and production system, as well as on pooled data across seasons to assess overall genotypic performance. Best Linear Unbiased Predictors (BLUPs) were estimated to obtain stable genotypic values by accounting for environmental variance and genotype × environment interactions. Pearson’s correlation coefficient amongst traits were computed using BLUP values.

Genetic diversity parameters including number of alleles (Na), number of effective alleles (Ne), observed heterozygosity (Ho), expected heterozygosity (He), were estimated using GenAlEx 6.5 software (Peakall and Smouse, 2012). Polymorphism information content (PIC) was calculated using the standard formula: PIC = 1 − Σ(pi²), where *pi* is the frequency of the *i*th allele (Botstein et al., 1980).

Neighbor-joining (NJ) cluster analysis was performed using DARwin 6.5 software based on SSR marker-derived dissimilarity matrices (Perrier, 2006; Raza et al., 2020). Bootstrap analysis with 10,000 resamplings was performed to assess the robustness and consistency of genotype clustering. The NJ tree was visualised and annotated using the Interactive Tree of Life (iTOL) platform, and bootstrap support values for selected nodes were displayed (Letunic and Bork, 2021).

For Marker-Trait Association (MTA), single marker analysis (SMA) using single-factor ANOVA was employed. Marker alleles were treated as independent factors and tested against corresponding grain Fe and Zn BLUP values following the procedures of Raza et al. (2020). Significant associations were determined based on p-values, and the coefficient of determination (R^2^) was used to estimate the proportion of phenotypic variation explained by individual markers.

Given the targeted nature of the study, involving 80 genotypes and 35 SSR markers, SMA was adopted as a parsimonious approach for detecting marker-trait associations. Although advanced models such as General Linear Models (GLM) and Mixed Linear Models (MLM) are widely used, their effectiveness depends on adequate genome-wide marker coverage for reliable estimation of population structure (Q) and kinship (K) matrices. In the present study, the relatively low marker density may limit the robustness of such model-based approaches (Breseghello and Sorrells, 2006). Therefore, SMA was considered appropriate for the initial detection of marker-trait associations. Significance was assessed at *p* < 0.05.

## 3 Results

### 3.1 Analysis of variance for grain Fe and Zn concentration

The pooled analysis of variance (ANOVA) showed highly significant differences (p < 0.01) amongst genotypes for both grain Fe and Zn concentration, indicating the presence of substantial genetic variability within the studied germplasm (**Table 1**). The effect of production system (E) was significant for grain Fe but non-significant for Zn, suggesting that Fe accumulation is influenced by management conditions, whereas Zn accumulation remained relatively stable across production systems.

**Table 1:** Pooled mixed-model analysis of variance (ANOVA) for grain Fe and Zn concentrations in rice genotypes evaluated under organic and inorganic production systems.

Source of variation	df	p-value	
		Fe	Zn
Genotype (G)	79	<0.01**	<0.01**
Environment (E)	1	<0.01**	0.54ns
Year (Y)	1	<0.01**	0.014*
G×E	79	<0.01**	<0.01**
G×Y	79	<0.01**	<0.01**
E×Y	1	0.08ns	<0.01**
G×E×Y	79	<0.01**	<0.01**

Significance levels are denoted as ns (non-significant), *p < 0.05 and **p < 0.01.

Season (year) effects were significant for both Fe (p < 0.01) and Zn (p < 0.05), indicating that seasonal variation affected micronutrient concentration. The genotype × environment (G × E) and genotype × year (G × Y) interactions were highly significant (p < 0.01) for both traits, showing that genotypes responded differently across production systems and seasons.

The environment × year (E × Y) interaction was non-significant for Fe but significant for Zn, indicating that Zn concentration was influenced by the combined effect of year and production system. Furthermore, the three-way interaction (G × E × Y) was also highly significant (p < 0.01) for both micronutrients, indicating that genotype performance is dependent on the specific combination of season and production system.

### 3.2 Variability and frequency distribution of grain Fe and Zn concentration

**Figure 1:**
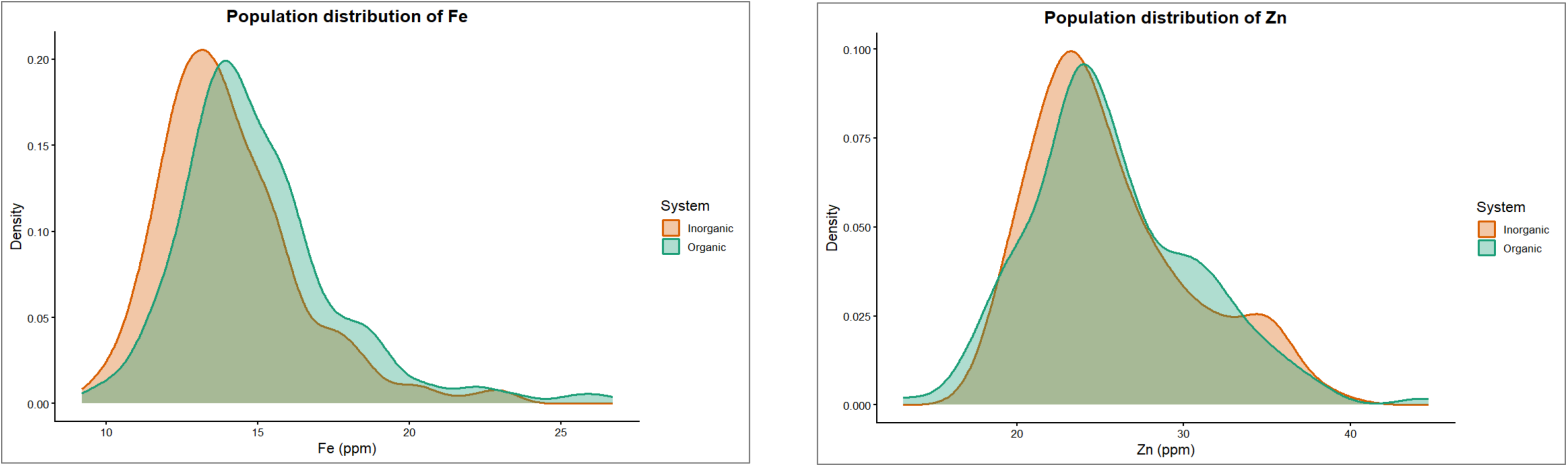
Population distribution of grain iron (Fe) and zinc (Zn) concentrations in rice genotypes under organic and inorganic production systems.

Grain Fe and Zn concentrations of 80 rice genotypes grown under organic and inorganic production systems during 2023 and 2024 were estimated using ED-XRF analysis. The variation observed in micronutrient concentration (**Table 2**; **Figure 1**) is supported by the significant effects observed in ANOVA.

**Table 2:** Descriptive statistics of grain Fe and Zn concentrations (mg kg^-1^) in rice genotypes across years under organic and inorganic production systems.

Grain micronutrient	Production system	Year	Mean ± SE	Coefficient of variation (%)	Minimum	Maximum
Fe	Organic	2023	15.47 ± 0.19	16.25	10.3	23.7
		2024	14.56 ± 0.22	19.21	9.3	26.8
		Pooled	15.01 ± 0.15	17.94	9.3	26.7
	Inorganic	2023	14.42 ± 0.18	16.11	9.8	23.2
		2024	13.70 ± 0.18	16.91	9.2	22.9
		Pooled	14.06 ± 0.12	16.67	9.2	23.2
Zn	Organic	2023	26.16 ± 0.38	18.54	16.5	38.6
		2024	25.35 ± 0.41	20.62	13.2	44.7
		Pooled	25.75 ± 0.28	19.61	13.2	44.7
	Inorganic	2023	24.75 ± 0.29	15.13	18.5	35.7
		2024	26.59 ± 0.44	20.78	17.5	40.0
		Pooled	25.67 ± 0.27	18.71	17.5	40.0

Grain Fe concentration ranged from 9.2 mg kg^-^¹ to 23.2 mg kg^-^¹ under inorganic conditions and from 9.3 mg kg^-^¹ to 26.7 mg kg^-^¹ under organic conditions. The pooled mean grain Fe concentration was higher in organic (15.01 ± 0.15 mg kg^-^¹) than inorganic (14.06 ± 0.12 mg kg^-^¹) conditions. The coefficient of variation (CV) for Fe concentration was moderate, ranging from 16.67% in inorganic to 17.94% in organic systems. The frequency distribution of Fe concentration was nearly normal with slight right skewness in both systems. However, the organic system showed a slightly wider distribution with more genotypes towards higher Fe values.

Similarly, Zn concentration varied considerably amongst genotypes, ranging from 17.5 mg kg^-^¹ to 40.0 mg kg^-^¹ under inorganic conditions and from 13.2 mg kg^-^¹ to 44.7 mg kg^-^¹ under organic conditions. The mean Zn concentration was similar between inorganic (25.67 ± 0.27 mg kg^-^¹) and organic (25.75 ± 0.28 mg kg^-^¹) systems. The CV for Zn concentration was 18.71% in inorganic and 19.61% in organic systems, indicating moderate variability. The distribution was approximately normal with moderate right skewness, and the organic system displayed a slightly wider spread.

Overall, the observed variability and distribution indicate that grain Fe and Zn concentrations are quantitative traits, suggesting that there is sufficient genetic variation to support effective selection under both production systems.

### 3.3 Genetic variability parameters across seasons and production systems

The genetic variability parameters estimated using REML-based analysis showed moderate to high variability for both Fe and Zn concentration across seasons and production systems (**Table 3**). Genetic variability parameters were estimated separately for each season under both production systems, followed by a pooled analysis across seasons. The PCV was slightly higher than GCV, indicating influence of environment on trait expression.

**Table 3:** Estimates of genetic variability parameters for grain Fe and Zn concentrations in rice genotypes across years under organic and inorganic production systems.

Grain micronutrient	Production system	Year	GCV (%)	PCV (%)	H2 (%)	GA	GAM (%)
Fe	Organic	2023	16.10	16.19	98.7	5.09	32.95
		2024	18.81	19.04	97.6	5.57	38.27
		Pooled	15.91	16.99	87.7	4.61	30.70
	Inorganic	2023	15.95	16.05	98.7	4.71	32.63
		2024	16.69	16.82	98.4	4.67	34.11
		Pooled	14.28	15.55	84.3	3.79	27.01
Zn	Organic	2023	18.30	18.45	98.4	9.78	37.39
		2024	20.10	20.39	97.2	10.35	40.81
		Pooled	17.36	18.56	87.5	8.61	33.45
	Inorganic	2023	14.79	14.98	97.5	7.44	30.08
		2024	20.52	20.68	98.5	11.15	41.94
		Pooled	13.19	16.21	66.2	5.68	22.11

GCV, genotypic coefficient of variation; PCV, phenotypic coefficient of variation; H², broad-sense heritability; GA, genetic advance; GAM, genetic advance as percent of mean.

For grain Fe, GCV ranged from 14.28% to 18.81% and PCV ranged from 15.55% to 19.03%. Broad sense heritability (H²) was high under most conditions, and GAM ranged from 27.0% to 38.27%. The concurrence of high heritability and GAM suggests that Fe concentration is likely governed by additive gene action, making selection for this trait highly effective.

For grain Zn, GCV ranged from 13.19% to 20.52% and PCV ranged from 14.98% to 20.68%. While H² was high in individual seasons, it was reduced under pooled analysis (66.2%), indicating the influence of G × E interaction. GAM for Zn ranged from 22.11% to 41.93%, indicating scope for improvement through phenotypic selection. Overall, the reduction in heritability under pooled analysis, particularly for Zn, suggests that environmental interactions significantly influence trait expression.

### 3.4 Effect of production system and grain colour on grain micronutrient concentration

**Figure 2:**
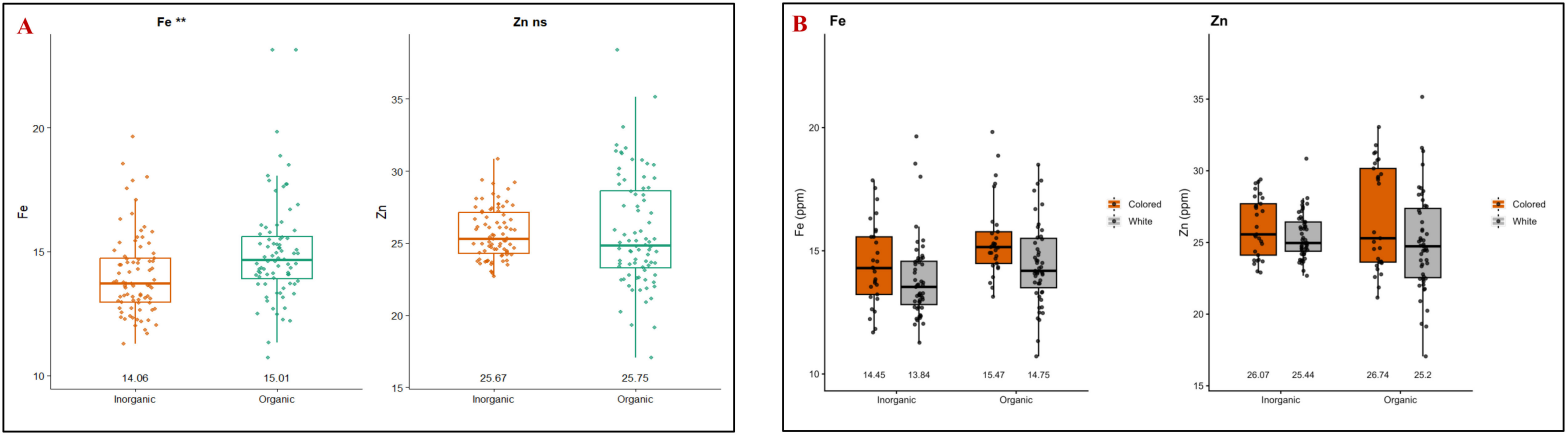
Influence of production system and grain colour on grain Fe and Zn concentrations in rice genotypes. (A) Effects of organic and inorganic production systems on grain Fe and Zn concentrations. (B) Grain colour-wise distribution of Fe and Zn concentrations under contrasting production systems. The symbol ** indicates significance at P < 0.01, whereas ns indicates non-significant differences (P ≥ 0.05).

The influence of the production system on grain Fe and Zn concentration is presented in **Figure 2A**. Grain Fe differed significantly (p < 0.01) between production systems, with organic cultivation yielding higher mean Fe concentration (15.01 mg kg^-^¹) than inorganic conditions (14.06 mg kg^-^¹). In contrast, Zn concentration remained statistically comparable between organic (25.75 mg kg^-^¹) and inorganic (25.67 mg kg^-^¹) systems, indicating no significant effect of production system on Zn concentration.

The effect of grain colour on Fe and Zn concentration is shown in **Figure 2B**. Coloured rice genotypes showed slightly higher mean Fe concentration than white rice under both inorganic (14.45 vs 13.84 mg kg^-^¹) and organic (15.47 vs 14.75 mg kg^-^¹) systems. A similar trend was observed for Zn, where coloured genotypes showed slightly higher mean values than white rice under both inorganic (26.07 vs. 25.44 mg kg^-^¹) and organic (26.74 vs. 25.32 mg kg^-^¹) conditions. Notably, the coloured genotypes also displayed a wider range of variation for both nutrients compared to the white rice group.

### 3.5 Superior grain Fe and Zn genotypes

**Figure 3:**
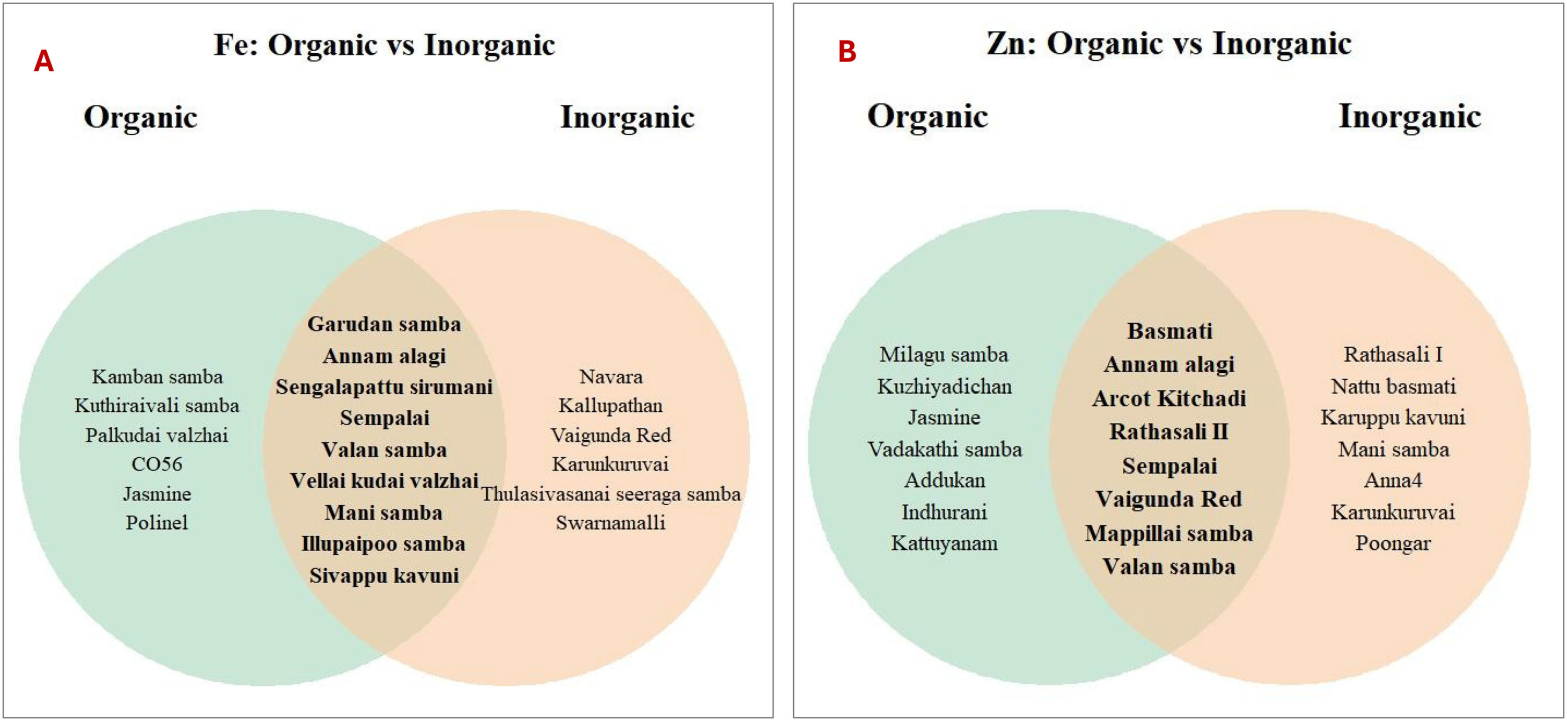
Venn diagrams showing the overlap of top 15 rice genotypes for grain Fe and Zn concentrations under organic and inorganic production systems based on BLUP estimates. **(A)** Top 15 Fe genotypes **(B)** Top 15 Zn genotypes.

Based on BLUP-based ranking, the top 15 rice genotypes for grain Fe and Zn concentration were identified for both organic and inorganic production systems (**Table 4**; **Figure 3**). Under organic conditions, the top-performing genotypes for Fe ranged from 16.07 to 23.13 mg kg^-1^, with Kamban samba and Garudan samba exhibiting the highest concentrations. In contrast, the top Fe-rich genotypes under inorganic conditions showed relatively lower Fe concentration, ranged from 15.36 to 19.64 mg kg^-1^, led by Garudan samba and Mani samba. Among the top fifteen genotypes for grain Fe, Garudan Samba, Annam Alagi, Sempalai, Valan Samba, Vellai kudai valzhai, Mani samba, Illupaipoo samba, Sivappu kavuni and Sengalapattu sirumani showed consistent performance across both production systems.

**Table 4:** Ranking of top-performing rice genotypes for grain Fe and Zn concentrations under organic and inorganic production systems.

Organic production system				Inorganic production system			
Top 15 genotypes for grain Fe							
Rank	Genotype	Grain colour	Fe (mg kg-1)	Rank	Genotype	Grain colour	Fe (mg kg-1)
1	Kamban samba	W	23.13	1	Garudan samba	W	19.64
2	Garudan samba	W	23.05	2	Mani samba	W	18.54
3	Annam alagi	C	19.82	3	Illupaipoo samba	W	18.01
4	Kuthiraivali samba	C	18.86	4	Navara	C	17.86
5	Sengalapattu sirumani	W	18.50	5	Valan samba	C	17.55
6	Sempalai	W	18.07	6	Annam alagi	C	17.09
7	Palkudai valzhai	W	17.85	7	Sivappu kavuni	C	16.53
8	Valan samba	C	17.72	8	Kalluputhan	C	16.31
9	CO56	W	17.72	9	Sengalapattu sirumani	W	15.98
10	Vellai kudai valzhai	C	17.62	10	Vellai kudai valzhai	C	15.85
11	Mani samba	W	17.44	11	Sempalai	W	15.79
12	Illupaipoo samba	W	16.89	12	Vaigunda Red	C	15.57
13	Jasmine	W	16.69	13	Karunkuruvai	C	15.57
14	Sivappu kavuni	C	16.18	14	Thulasivasanai seeraga samba	W	15.42
15	Polinel	W	16.07	15	Swarna malli	W	15.36
Top 15 genotypes for grain Zn							
Rank	Genotype	Grain colour	Zn (mg kg-1)	Rank	Genotype	Grain colour	Zn(mg kg-1)
1	Basmati	W	38.38	1	Basmati	W	30.84
2	Milagu samba	W	35.15	2	Valan samba	C	29.39
3	Annam alagi	C	33.05	3	Annam alagi	C	29.22
4	Kuzhiyadichan	C	27.56	4	Vaigunda Red	C	29.13
5	Arcot Kitchadi	W	31.60	5	Rathasali II	C	28.75
6	Jasmine	W	31.37	6	Mappillai samba	C	28.40
7	Rathasali II	C	31.28	7	Sempalai	W	28.21
8	Sempalai	W	31.28	8	Rathasali I	C	28.10
9	Vadakathi samba	C	30.81	9	Nattu basmati	W	28.09
10	Vaigunda Red	C	30.74	10	Arcot Kitchadi	W	27.86
11	Adukkan	C	30.52	11	Karuppu kavuni	C	27.70
12	Indhurani	W	30.43	12	Mani samba	W	27.70
13	Mappillai samba	C	30.16	13	Anna4	W	27.61
14	Valan samba	C	29.77	14	Karunkuruvai	C	27.55
15	Kattuyanam	C	29.58	15	Poongar	C	27.51

Values represent pooled mean grain iron (Fe) or zinc (Zn) concentration (mg kg^-1^) across two seasons (Kharif 2023 and 2024) under organic and inorganic production systems. Rankings are based on best linear unbiased predictor (BLUP) values estimated within each production system. Grain colour: W, white; C, coloured.

For grain Zn concentration, higher values were observed under organic conditions (29.58 - 38.38 mg kg^-1^) compared to inorganic system (27.51 - 30.84 mg kg^-1^), with Basmati ranking highest in both systems. Genotypes including Basmati, Annam alagi, Arcot Kitchadi, Rathasali II, Sempalai, Vaigunda Red, Mappillai samba and Valan samba were consistently identified as top performers across both systems.

Notably, Annam alagi, Sempalai and Valan samba were identified as superior for both Fe and Zn concentration across environments, indicating their stable performance for multi-nutrient biofortification. The overlap of genotypes between organic and inorganic systems was moderate, at 60% for Fe and 53.3% for Zn; this confirm that whilst some genotypes are stable, and the production system significantly influences ranking patterns (**Figure 3**).

### 3.6 Relationship between grain Fe and Zn concentration

**Figure 4:**
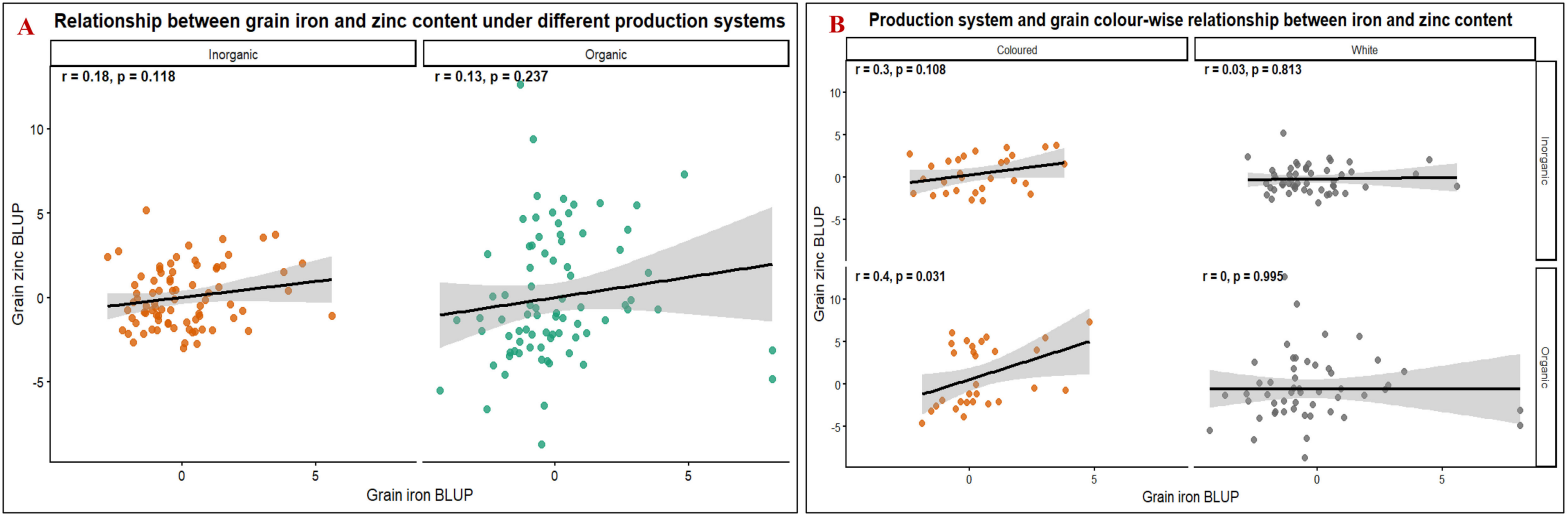
Relationship between grain iron (Fe) and zinc (Zn) concentrations in rice genotypes under contrasting production systems and grain colour classes. **(A)** Association between grain Fe and Zn BLUP values under inorganic and organic production systems. **(B)** Grain colour-wise relationship between Fe and Zn BLUP values within coloured and white rice genotypes across production systems. Solid lines represent fitted linear regressions with shaded 95% confidence intervals; Pearson's correlation coefficients (r) and significance levels are indicated.

The relationship between grain Fe and Zn concentration was examined using scatter plot and Pearson’s correlation coefficients based on BLUP values (**Figure 4A**). For the total population, grain Fe and Zn exhibited a weak and non-significant positive association under both inorganic (r=0.18) and organic (r=0.13) production systems suggesting the absence of a strong linear relationship between these two micronutrients when considering all genotypes together.

When stratified by grain colour and production system (**Figure 4B**), coloured rice genotypes showed a positive association between grain Fe and Zn concentration in both environments. This association was moderate and statistically significant under organic condition (r = 0.40, p < 0.05), whereas under inorganic conditions the relationship was positive but non-significant (r = 0.30, p = 0.108). In contrast, white rice genotypes did not exhibit any significant association under either inorganic (r = 0.03, p = 0.813) or organic (r = 0.00, p = 0.995) production systems, indicating independent accumulation of the two micronutrients.

**Figure 5:**
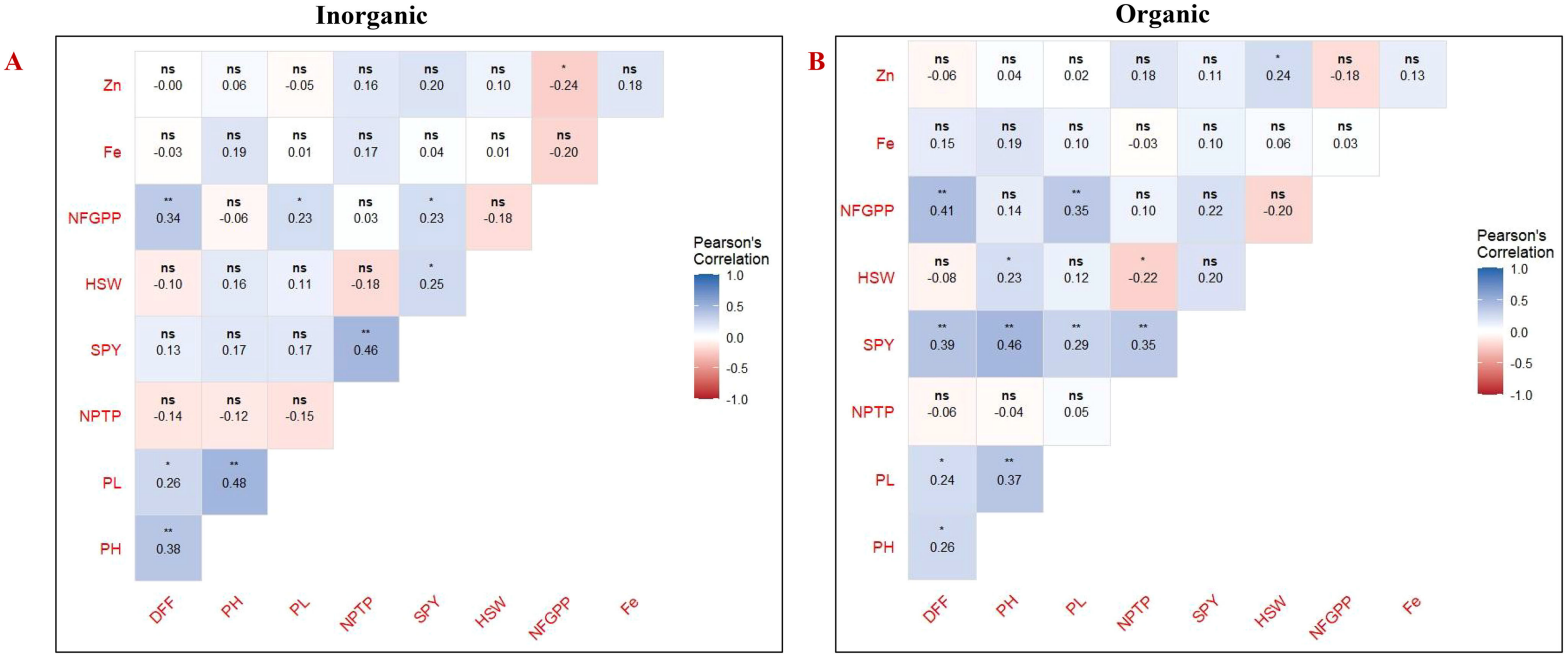
Correlation heat maps showing the relationships between grain micronutrient concentrations (Fe and Zn) and key biometric traits in rice under **(A)** inorganic and **(B)** organic production systems. The colour scale represents the strength and direction of correlation, with r values displayed in each cell. Asterisks indicate significance levels: * P < 0.05 and ** P < 0.01, whereas ns indicates a non-significant correlation (P ≥ 0.05).

Furthermore, correlation analysis was performed to evaluate whether enhancement of grain Fe and Zn is associated with key agronomic traits (**Figure 5**). Traits including DFF, PH, PL, NPTP, HSW and SPY, revealed consistently weak and non-significant associations (p > 0.05) across both production systems. These results indicate that increasing grain micronutrient concentration can likely be achieved without negatively impacting major agronomic performance or yield. To further support this observation, agronomic performance of genotypes showing overlapping high Fe and Zn is presented in **Supplementary Table 4**. These genotypes exhibited comparable performance for key agronomic traits, reinforcing the absence of a yield penalty and highlighting their suitability for biofortification breeding.

### 3.7 Marker diversity analysis using SSR marker

**Figure 6:**
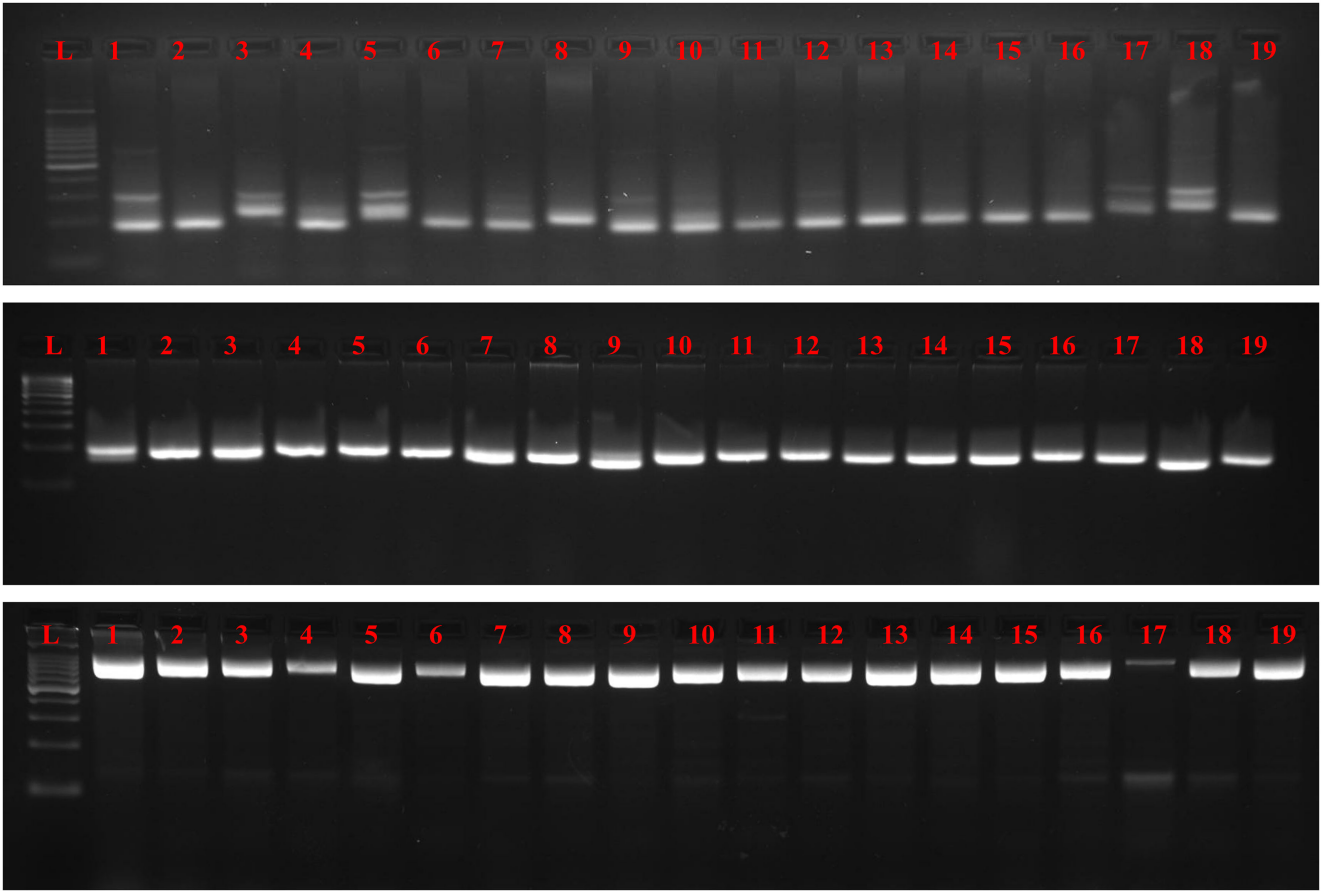
Representative gel images of different markers. **(A)** RM247, **(B)**RM 231 and **(c)** OsYSL2b. Lane L indicates the DNA ladder (100bp), and lanes 1–19 represent individual rice accessions (**Supplementary Table 1**).

DNA fingerprinting of 80 genetically diverse rice accessions was conducted using 32 previously reported Fe and Zn-associated SSR markers and 5 gene-specific markers. Representative agarose gel images illustrating banding patterns of some rice accessions given in **Figure 6** (Original gel images were given in **Supplementary Figures 5**–**7**). Summary statistics of the polymorphic markers used are presented in **Table 5**. Out of 37 markers, 30 (81.08%) were found to be polymorphic and collectively generated a total of 88 alleles, indicating substantial allelic variation amongst the rice genotypes.

**Table 5:** Genetic diversity parameters of SSR and gene-specific markers associated with grain Fe and Zn concentrations in rice.

Marker	Na	Ne	I	Ho	He	PIC
RM72	2	1.703	0.603	0.000	0.413	0.427
RM234	3	1.710	0.741	0.000	0.415	0.458
RM248	4	2.550	1.146	0.203	0.608	0.482
RM190	3	1.560	0.660	0.000	0.359	0.375
RM223	3	1.838	0.758	0.000	0.456	0.456
RM231	3	2.872	1.077	0.000	0.652	0.660
RM260	3	2.794	1.059	0.114	0.642	0.659
RM541	2	1.140	0.243	0.000	0.123	0.208
RM335	4	3.353	1.267	0.544	0.702	0.305
RM488	2	1.995	0.692	0.000	0.499	0.449
RM493	3	1.964	0.833	0.000	0.491	0.491
RM3412	4	2.430	1.062	0.000	0.588	0.588
RM247	5	2.820	1.211	0.203	0.645	0.561
RM205	4	2.035	0.874	0.000	0.509	0.533
RM237	2	1.501	0.516	0.013	0.334	0.354
RM447	2	1.422	0.473	0.013	0.297	0.062
RM517	3	1.649	0.626	0.050	0.393	0.343
RM243	2	1.133	0.234	0.000	0.117	0.117
RM259	2	1.311	0.400	0.000	0.237	0.237
RM110	2	1.822	0.643	0.013	0.451	0.439
RM152	2	1.809	0.639	0.350	0.447	0.036
RM339	4	2.198	0.985	0.075	0.545	0.482
RM3644	2	1.220	0.325	0.000	0.180	0.18
RM5607	2	1.223	0.328	0.000	0.182	0.202
RM7364	4	2.559	1.078	0.093	0.609	0.299
OsYSL2b	4	3.369	1.291	0.000	0.703	0.718
OsZIP3b	3	2.040	0.872	0.250	0.510	0.598
OsNRAMP7	3	2.542	1.004	0.000	0.607	0.654
OsMTP1a	3	2.109	0.901	0.000	0.526	0.526
OsNAS3f	3	2.446	0.989	0.000	0.591	0.584

Na, number of alleles; Ne, effective number of alleles; I, Shannon’s information index; Ho, observed heterozygosity; He, expected heterozygosity; PIC, polymorphism information content.

The number of alleles per locus varied from 2 to 5, with the highest number of alleles recorded for RM247, and an average of 2.93 alleles per locus. The Shannon’s information index (I) ranged from 0.234 to 1.291, reflecting a moderate to high degree of genetic diversity. Markers such as RM335, RM247 and OsYSL2b showed higher diversity values, indicating higher allelic richness and evenness. The observed heterozygosity (Ho) varied from 0.000 to 0.544, whilst the expected heterozygosity (He) varied from 0.117 to 0.703.

The Polymorphic Information Content (PIC) values varied from 0.036 (RM152) to 0.718 (OsYSL2b), with an average PIC value of 0.416, indicating moderate to high informativeness of the SSR markers used in this study. It is pertinent to note that the gene-specific markers, such as OsYSL2b, OsNRAMP7, and OsNAS3f showed higher PIC and diversity values, indicating their higher informativeness in assessing genetic variation.

### 3.8 Grouping of rice genotypes by phylogenetic analysis

**Figure 7:**
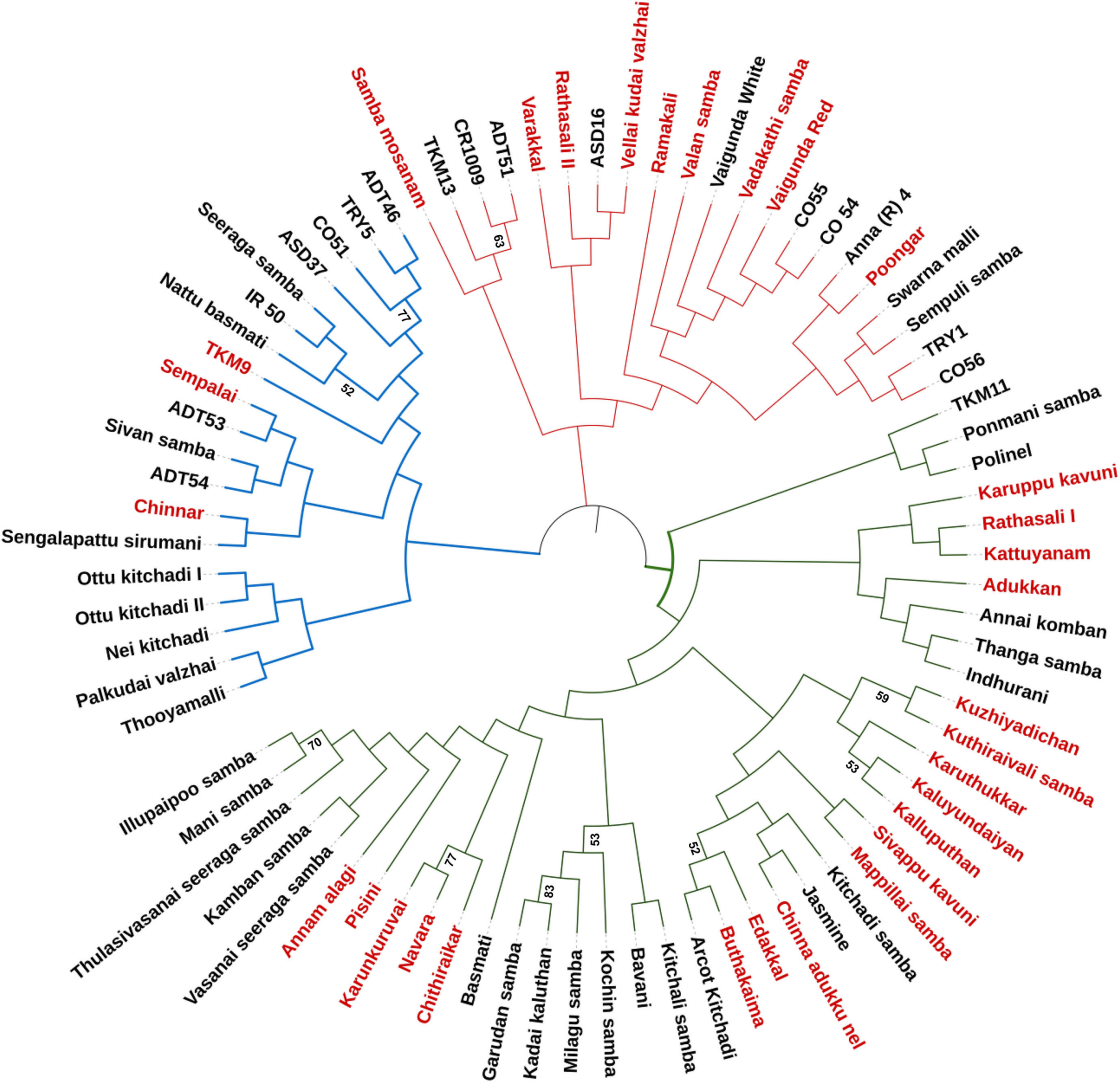
Neighbour-Joining (NJ) dendogram base on molecular marker data showing genetics relationships among rice genotypes. Number at nodes represent bootstrap support percentages (≥50%) from 10, 000 resampling; Genotype names highlighted in red represent coloured-grain rice genotypes, whereas those in black represent white-grain rice genotypes.

The Neighbor-Joining (NJ) dendrogram based on SSR marker data grouped the 80 rice genotypes into three major clusters, each further subdivided into multiple sub-clusters (**Figure 7**). The clustering pattern revealed a substantial admixture of traditional landraces and modern varieties, suggesting that genetic relatedness was not strictly associated with varietal status or pedigree. Several coloured-grain landraces formed partial sub-clusters; however, many were interspersed with white-grain genotypes. This suggests that grain pigmentation represents only partial genetic structuring within the broader germplasm.

Genotypes within the same sub-clusters showed relatively higher genetic similarity, whereas those grouped into different major clusters showed greater genetic divergence, consistent with the SSR-based genetic dissimilarity matrix. Bootstrap support values across the dendrogram ranged from 12% to 82%, reflecting variable levels of grouping consistency among the evaluated genotypes. For clarity, only bootstrap support values greater than 50% were displayed for selected nodes in the NJ dendrogram. Overall, the clustering pattern reflected the diverse and admixed genetic background of the rice panel.

### 3.9 Single marker analysis

Single-marker analysis (SMA) based on single-factor ANOVA was employed to identify associations between 30 polymorphic markers and grain Fe & Zn concentration under inorganic and organic production systems. Statistically significant marker-trait associations (p<0.05) were detected for 15 markers across the two environments (**Table 6**).

**Table 6:** Marker–trait associations for grain Fe and Zn concentrations under organic and inorganic production systems in rice.

Marker	CN	Inorganic		Organic	
		p-value	R2	p-value	R2
Grain Fe					
RM493	1	0.035	5.57	0.577	-
RM259	1	0.025	6.32	0.163	-
RM5607	2	0.019	6.99	0.004	10.36
OsYSL2b	2	0.072	-	0.015	7.3
RM231	3	0.021	6.69	0.047	5.02
RM335	4	0.039	5.44	0.462	-
RM234	7	0.031	6.08	0.089	-
RM248	7	0.043	5.13	0.045	5.04
RM223	8	0.106	-	0.048	4.93
RM339	8	0.067	-	0.022	6.53
RM205	9	0.001	13.8	0.125	-
RM260	12	0.182	-	0.037	6.22
OsNRAMP7	12	0.436	-	0.007	9.06
Grain Zn					
RM237	1	0.040	5.39	0.972	–
OsYSL2b	2	0.234	–	0.032	5.76
RM447	8	0.030	5.88	0.065	-

Markers showing significant associations (p ≤ 0.05) in at least one production system are presented, along with corresponding values across environments to facilitate comparison of marker effects. CN, Chromosome number. p-value – Significance of marker–trait association. R² - proportion of phenotypic variance explained by the marker. – indicates non-significant association or R² not estimated. Associations were identified separately under organic and inorganic production systems using single-marker analysis. Gene-specific markers are indicated by marker names beginning with “Os”.

Under inorganic production system, eight markers showed significant association with grain Fe concentration, explaining phenotypic variance (R^2^) ranging from 5.13% (RM248) to 13.8% (RM205). Among these, RM205 on chromosome 9 exhibited the strongest association with grain Fe concentration (p = 0.0008). Under organic management, a different set of markers was significantly associated with grain Fe concentration, with R^2^ values ranging from 4.93% (RM223) to 10.36% (RM5607). Notably, three markers, namely RM248, RM231 and RM5607, showed significant and stable association with grain Fe concentration under both management systems, indicating their potential for marker-assisted selection (MAS).

In contrast to grain Fe, fewer significant MTAs were identified for grain Zn concentration. Under inorganic conditions, significant associations were detected for RM237 and RM447, explaining 5.59% and 5.88% respectively. Under organic conditions, only the gene specific marker OsYSL2b showed significant association with grain Zn concentration, explaining 5.76% of the phenotypic variation.

Among the gene-specific markers evaluated, OsYSL2b located on Chromosome 2 showed significant association with both grain Fe (7.3%) and Zn (5.76%) under organic conditions. In addition, OsNRAMP7 (chromosome 12) was significantly associated with grain Fe (9.06%) specifically under the organic production system.

## 4 Discussion

Micronutrient malnutrition, especially Fe and Zn deficiency, remains a major concern in rice-based diets, necessitating the identification of nutritionally superior and agronomically suitable genotypes. While research has traditionally focused on enhancing grain Fe and Zn under conventional inorganic systems, the combined influence of production systems, seasonal variation, and genetic background remains under-explored. In this regard, the present study combines multi-season evaluation under organic and inorganic production systems with genetic variability analysis to determine the breeding potential of diverse rice genotypes.

### 4.1 Genetic variability and selection potential of grain Fe and Zn concentrations

The presence of genetic variation is the primary requirement for enhancing micronutrient levels through breeding. In the present study, the highly significant genotypic differences (**Table 1**) and wide phenotypic ranges (**Table 2**) confirmed the existence of substantial variability amongst the 80 rice genotypes. Such variability forms the basis for effective selection and long-term genetic improvement (Sanghamitra et al., 2022).

The observed variability was further supported by moderate to high GCV and PCV values across both systems (**Table 3**). The narrow gap between these parameters indicates that the phenotypic expression of micronutrient concentration is largely influenced by genetic factors, with a relatively lower environmental effect. The high h^2^ coupled with moderate to high GAM observed for Fe and Zn suggests that these traits are primarily governed by additive gene action. This confirms a strong potential for genetic improvement through direct phenotypic selection.

The wide genetic variability observed in the present study agrees with earlier reports (Garcia-Oliveira et al., 2018; Bollinedi et al., 2020; Islam et al., 2020; Raza et al., 2020). Notably, the higher variability and trait expression observed in traditional rice types, may be attributed to lower historical selection pressure for yield and grain quality during varietal development, allowing the retention of micronutrient-related alleles (Azeez et al., 2018).

### 4.2 Effect of production system and environmental interaction

The production system and seasonal variation played an important role in determining grain micronutrient concentration. Our results showed that (**Figure 2A**), grain Fe concentration was higher under organic production systems, whereas Zn concentration remained comparable across systems. This differential response indicates that Fe accumulation is more sensitive to management conditions, whereas Zn appeared to show comparatively lower sensitivity to production system.

The higher grain Fe concentration observed in organic systems is likely to be associated with improved nutrient availability driven by increased soil organic matter and microbial activity. In organic plots, increased synthesis of organic acids and siderophores may facilitate the formation of transportable iron chelates, thereby increasing root uptake (Cakmak et al., 2010; Rengel, 2015). In contrast, the stability of Zn across systems suggests a relatively regulated translocation of Zn to the rice grain. Similar trends have been reported where Zn accumulation showed relatively lower environmental influence compared to Fe (Impa et al., 2013; Nayak et al., 2023).

The highly significant G × E, G × Y and G × E × Y interactions (**Table 1**), underscore that genotypic performance for micronutrient density is highly context - dependent. This highlights the importance of evaluating genotypes under specific production systems and across seasons to identify stable performers. Despite the non-significant main effect of production system for Zn, the significant interaction effects suggest that both traits are influenced by genotype × environment interactions. The reduction in pooled heritability for Zn (Section 3.3) further supports the influence of environmental interactions on trait expression. Overall, these results indicate that Fe accumulation is more responsive to environmental conditions, whereas Zn shows relatively stable expression across production systems, although both traits are influenced by interaction effects.

### 4.3 Influence of grain pigmentation on micronutrient accumulation

Grain colour is a well-documented factor influencing micronutrient accumulation in rice. In the present study, coloured (pigmented) rice genotypes consistently showed higher grain Fe and Zn concentration than white rice genotypes across both production systems (**Figure 2B**). This trend indicates that pigmentation in rice grain is associated with enhanced micronutrient loading. The results of the current study agree with previous studies that reported two- to three-folds higher grain Fe and Zn concentration in red and brown rice compared to white rice varieties (Graham et al., 2001; Nachimuthu et al., 2014; Anadhu et al., 2025).

The nutritional superiority of pigmented rice may be attributed to distinct structural and biochemical characteristics. As a substantial amount of Fe and Zn in the grain is localised in the aleurone and outer bran layers, brown (unpolished) rice naturally retains higher micronutrient levels (Bollinedi et al., 2020; Raza et al., 2020). Furthermore, pigmented rice genotypes accumulate higher concentrations of secondary metabolites, such as phenolic compounds, flavonoids and anthocyanins, which act as natural chelators. These compounds can bind and sequester Fe and Zn within the grain matrix, preventing leaching and enhancing retention. Additionally, the aleurone layer is often more prominent in traditional landraces than in modern cultivars, further contributing to their nutritional superiority (Gregorio, 2002; Neeraja et al., 2018; Kaur et al., 2025).

From a plant breeding perspective, the higher variability observed in red and brown rice offers a broader genetic base for selection. Interestingly, high Fe and Zn concentrations have also been linked to aromatic varieties such as Jasmine and Basmati (Graham et al., 2001). The presence of duplicate epistatic interactions in the expression of grain Fe and Zn (Kumar et al., 2020) can be effectively exploited through recombination breeding, coupled with selection in later generations, to simultaneously increase both minerals.

The enhanced micronutrient composition in coloured landraces may also stem from divergent selection pressures during domestication. Modern varieties have undergone intense selection for yield and milling quality whereas traditional landraces likely conserved ancestral alleles for mineral deposition that were lost in the “bottleneck” of modern white rice breeding. The variation in mineral concentration between aromatic and non-aromatic, fine and coarse grain types, further supports the role of grain characteristics in determining nutrient composition (Raza et al., 2020; Verma and Srivastav, 2017).

The relatively higher micronutrient concentration in these coloured landraces highlights their potential as valuable donor parents in biofortification breeding programs (Swamy et al., 2018). The incorporation of these nutritionally rich genotypes into breeding programs will enable the development of “double-plus” varieties that combine high nutritional value with desirable agronomic traits for both organic and low-input systems.

### 4.4 Identification of superior genotypes for grain Fe and Zn

Considering the observed effects of production system and grain colour on micronutrient accumulation, the identification of superior genotypes for grain Fe and Zn was further examined. The top-ranking genotypes (**Figure 3**) under organic and inorganic production systems emphasise the presence of large exploitable variability for micronutrient accumulation in rice. The distinct sets of top-performing genotypes identified across environments further confirm the strong influence of production system on micronutrient expression and genotype ranking.

The overlap of top-ranking genotypes between production systems was moderate, with 60% for Fe and 53.3% for Zn, indicating that although a subset of genotypes showed stable performance, environmental conditions still influenced ranking patterns. The moderate overlap between the top Fe- and Zn-rich genotypes further supports the partially independent genetic control of these micronutrients (Swamy et al., 2018), suggesting that whilst some pathways for mineral loading may be shared, others are nutrient-specific.

In the present study, Kamban Samba and Garudan Samba exhibited high grain Fe concentration under organic and inorganic conditions respectively, indicating their potential as reliable Fe donors. Similarly, Basmati showed stable and superior grain Zn concentration across environments, highlighting its suitability as a Zn donor. Notably, genotypes such as Annam alagi, Valan samba, and Sempalai exhibited superior performance for both traits and across environments, suggesting their potential utility as donor parents for developing nutritionally enhanced rice varieties. Similar reports highlighting the contribution of traditional rice landraces to micronutrient improvement further reinforce their value in biofortification breeding programmes (Raza et al., 2019a; Anusha et al., 2021).

### 4.5 Relationship between grain Fe and Zn concentration and agronomic traits

Understanding the relationship between grain micronutrient concentration and key agronomic traits is essential for effective biofortification, as both favourable and unfavourable linkages influence selection decisions. In the present study (**Figure 4**), the weak and non-significant association between grain Fe and Zn concentration across the entire panel suggests that the accumulation of these micronutrients is not always tightly coupled across diverse genetic backgrounds and environments. This indicates that Fe and Zn homeostasis may be governed by partially independent physiological mechanisms (Senguttuvel et al., 2023).

Notably, when the data were stratified, a significant positive correlation between grain Fe and Zn concentration was observed specifically in coloured rice genotypes under organic production system (r = 0.40, p < 0.05). Such genotype- and environment-dependent association suggests that coordinated accumulation of Fe and Zn may occur only in certain genetic backgrounds and under favourable conditions. Earlier studies have reported moderate positive correlations in traditional rice varieties, aromatic rice, and landraces, whilst weak or inconsistent associations were observed in diverse germplasm panels (Cantila and Quitel, 2020; Lee et al., 2020; Raza et al., 2020; Anadhu et al., 2025). These findings indicate that the relationship between Fe and Zn varies depending on genotype and environment.

A critical finding was the absence of strong negative correlations between grain micronutrients and key agronomic traits, including grain yield (**Figure 5**). This is a vital observation for breeding, as it suggests that increasing nutrient density is unlikely to compromise productivity. This aligns with the findings of Pandey et al., 2019, who reported no significant associations between grain yield and micronutrient concentration. Furthermore, the weak associations observed in the present study suggest that the negative correlations sometimes reported in modern cultivars (Dixit et al., 2019) may be influenced by past selection pressure for yield rather than strict physiological constraints. The lack of detectable trade-offs in our study reinforces the potential of biofortification-oriented breeding. As highlighted by (Senguttuvel et al., 2023), the use of high-Zn traditional rice varieties in pre-breeding programs can help overcome such limitations.

### 4.6 Marker informativeness and allelic diversity

The SSR-based molecular diversity analysis revealed substantial allelic diversity amongst the 80 rice genotypes evaluated in this study. The high percentage of polymorphic markers (81.08%) and the average number of alleles per locus (2.93) are comparable with earlier diversity studies in rice (Pradhan et al., 2020; Raza et al., 2020). The relatively lower allelic richness (6.77 alleles per locus) observed in the study compared to earlier reports (Vanlalsanga et al., 2019), may be attributed to the use of trait-linked and gene-specific markers rather than genomic-wide neutral SSR markers. Similar trends have been observed in micronutrient-focused studies, where functional or trait-linked markers captured relatively lower allelic diversity but remain relevant for target traits (Kumari et al., 2019).

The moderate to high Shannon’s information index (I) values further confirm the appreciable allelic variation within this panel. The lower H_o_ compared to H_e_ across most loci is consistent with the self-pollinated nature of rice, reflecting the higher homozygosity expected in traditional landraces.

The average PIC value (0.416), with ten marker exceeding PIC values of 0.5, indicates that the selected markers are highly informative for genetic characterisation. Comparable PIC values have also been reported in earlier rice studies (Kumari et al., 2019; Raza et al., 2020; Zulfiqar et al., 2023). Notably, the gene-specific markers OsYSL2b exhibited the highest PIC (0.718), indicating its effectiveness in differentiating genotypes based on functional variation. High level of polymorphism has also been previously documented in genomic regions associated with key metal transporter genes families including ZIP (Zrt- and Irt- like protein), YSL (Yellow Stripe-Like), NRAMP (Natural Resistance- Associated Macrophage Protein), VIT (Vacuolar Iron Transporter) and NAS (Nicotianamine Synthase) (Nachimuthu et al., 2014; Kumari et al., 2018; Neeraja et al., 2018). The substantial allelic variation observed in candidate genes such as OsNRAMP5 and OsYSL2b underscores their role in capturing the genetic basis of grain micronutrient accumulation. The molecular diversity detected amongst the evaluated genotypes highlights their potential utility as genetic resources for biofortification programmes.

### 4.7 Genetic relationships inferred by cluster analysis

The NJ dendrogram (**Figure 7**) revealed a highly diverse genetic landscape within the rice panel by grouping the genotypes into three major clusters with multiple sub-clusters, characterised by considerable intermixing of landraces and modern varieties. This absence of distinct grouping based on breeding history indicates a shared genetic background amongst several genotypes. These findings are consistent with earlier reports documenting shared ancestry and gene flow between traditional and improved rice germplasm (Raza et al., 2020; Upadhyay et al., 2011).

Several coloured-grain landraces exhibited partial clustering within certain sub-clusters but were also distributed across different clusters, indicating that grain colour alone does not correspond directly with overall genetic structure. Genotypes grouped within the same sub-clusters exhibited relatively higher genetic similarity, whereas those belonging to different major clusters showed greater genetic divergence, consistent with the SSR-based dissimilarity matrix.

The clustering pattern did not show a clear association with grain Fe and Zn concentration under different production systems. This is expected, as these traits are quantitatively inherited and influenced by multiple loci and genotype × environment interactions. Furthermore, the use of trait-associated and gene-specific SSR markers capture variation at specific loci associated with micronutrient traits rather than overall genome-wide relatedness.

Bootstrap-supported NJ clustering provided additional support for several genotype groupings, although support values varied across clusters. The relatively lower bootstrap support observed for certain major clusters may be attributed to the admixed nature of the germplasm panel, shared ancestry among landraces and modern varieties, and the weak population stratification detected within the panel. Similar patterns of moderate clustering support have been reported previously in SSR-based rice diversity studies involving genetically diverse germplasm collections (Raza et al., 2020; Upadhyay et al., 2011). The observed weak population structure is advantageous for marker–trait association studies, as low to moderate stratification can reduce the likelihood of false-positive associations (Kumari et al., 2018, 2025).

### 4.8 Marker trait association for grain Fe and Zn concentration

The MTAs identified using single marker analysis provided insights into the genomic regions governing Fe and Zn accumulation. The identification of 15 significant MTAs across inorganic and organic production systems confirms the complex, polygenic nature of grain micronutrient concentration in rice, as earlier suggested in QTL mapping studies (Kumari et al., 2018; Raza et al., 2020).

Individual markers in this study explained approximately 5 to 14% of the phenotypic variance (R^2^), a range typical for complex quantitative traits where multiple small-effect loci contribute to the overall phenotype (Gregorio et al., 2000; Garcia‐Oliveira et al., 2009; Swamy et al., 2018). This indicates the suitability of SSR-based single-marker analysis for preliminary identification of genomic regions associated with grain micronutrient traits.

Notably, a higher number of significant MTAs were detected for grain Fe than for Zn, a trend consistent with the higher environmental sensitivity observed for Fe in our phenotypic results (Section 3.1). The presence of partially distinct sets of markers across different production systems, suggests genotype × environment interactions controlling grain micronutrient concentration. Environment-specific QTLs and MTAs have been documented in biparental and association mapping studies under different nutrient management conditions (Swamy et al., 2018; Islam et al., 2020).

The stable markers identified across both production systems - RM231 (Chr 3), RM248 (Chr 7), and RM5607 (Chr 2), are highly valuable for marker-assisted selection (MAS) as they represent the genomic regions that contribute consistently to grain Fe accumulation irrespective of management conditions (Swamy et al., 2021).

The significant associations observed for gene specific markers, such as OsYSL2b and OsNRAMP7, suggest their potential relevance in micronutrient accumulation. These two markers are derived from well-characterised metal transporter gene families. Members of the NRAMP family, are involved in the transport of divalent metal ions such as Fe and Mn, whereas members of the YSL family play a role in the transport of Fe and Zn complexed with nicotianamine during long distance translocation and grain loading. The Observed association of OsNRAMP7 with Fe and OsYSL2b with both Fe and Zn under organic conditions is consistent with their reported biological functions. Previous studies also reported co- localisation of Fe and Zn related QTLs with members of YSL, NRAMP, and NAS gene families (Anuradha et al., 2012; Kumari et al., 2018, 2019; Calayugan et al., 2020). However, further functional validation is required to confirm the precise role of these genes in micronutrient accumulation under different production systems.

While the present study successfully identified several Marker–trait associations for grain Fe and Zn content, the use of single marker analysis has inherent limitations. The approach does not account for population structure or marker interactions, which may result in spurious associations. In addition, the relatively limited number of genotypes (80) and marker density (35 SSR markers) may affect the resolution and power of association detection. Therefore, the identified associations should be considered as preliminary.

Nevertheless, these findings provide a useful foundation for future studies. Further research involving larger and more diverse populations, higher marker density, and validation across multiple environments and production systems will be essential to confirm and refine these associations for effective application in biofortification breeding.

### 4.9 Linking elite genotypes with marker–trait associations

The elite genotypes identified through BLUP-based ranking provide a biological context for the significant MTAs detected in the study. Several genotypes that ranked amongst the top performers for grain Fe or Zn under organic conditions correspond to environments where gene-specific markers such as OsYSL2b and OsNRAMP7 showed significant associations. This concordance suggests the relevance of these loci and aligns with earlier reports demonstrating consistency between phenotypic superiority and molecular associations for grain micronutrient traits (Kumari et al., 2018, 2019).

The environment-specific superiority of certain genotypes, together with the detection of management-dependent MTAs, indicates the influence of genotype × management (G × M) interactions on micronutrient accumulation. Genotypes showing stable performance across environments and associated with markers such as RM231, RM248 and RM5607, represent the promising candidates for marker-assisted biofortification breeding with broad adaptation.

In contrast, genotypes showing better performance specifically under organic conditions and linked to environment-responsive gene-based markers may be more suitable for targeted breeding aimed at organic or low-input production systems. Overall, the integration of phenotypic ranking with molecular markers results strengthens confidence in the identified donor genotypes and provides a practical framework for their utilisation in rice biofortification breeding.

## 5 Conclusion

Micronutrient malnutrition resulting from Fe and Zn deficiency is a persistent problem in rice-based diets. The present study demonstrated significant phenotypic and genetic variation for grain Fe and Zn concentration amongst rice genotypes under organic and inorganic production systems. Significant genotype, environment, and interaction effects further highlighted the influence of production systems and seasons on micronutrient expression. Coloured rice genotypes showed consistently high grain Fe concentration across production systems, highlighting their importance as valuable genetic resources for micronutrient improvement.

The weak and non-significant associations observed between grain micronutrient concentration and key agronomic traits, including grain yield, indicate that improvement of Fe and Zn is achievable without compromising yield. This finding underscores the feasibility of simultaneous improvement of yield and micronutrient concentration in rice. The moderate genetic variability coupled with high heritability and genetic advance suggests good scope for selection. The moderate overlap of top-performing genotypes across production systems further indicates both stable and environment-specific genotype performance.

The molecular analysis revealed moderate to high allelic diversity and identified both stable and production system–specific marker–trait associations, including significant associations for gene-specific markers such as OsYSL2b and OsNRAMP7, supporting their role in grain micronutrient accumulation.

Overall, it was possible to identify elite genotypes as well as markers with biofortification potential using marker association analysis integrated with phenotypic performance. In addition, the differential performance of genotypes across organic and inorganic production systems emphasizes the need for production system–specific selection strategies. Collectively, these findings provide a robust genetic and phenotypic framework for the development of nutritionally enhanced rice varieties suited to sustainable and diverse cropping systems.

While the present study provides useful insights into marker–trait associations, it is important to note that single-marker analysis (SMA) was employed, which does not account for population structure or marker interactions as in more advanced models such as GLM or MLM. Therefore, the identified associations should be considered preliminary and require further validation using larger and more diverse populations, higher marker density, and advanced statistical models across different production systems.

## Data Availability

The original contributions presented in the study are included in the article/**Supplementary Material**. Further inquiries can be directed to the corresponding author.
