# Supplementary material for: Elite donors and stable marker–trait associations for grain iron and zinc biofortification in rice under organic and inorganic production systems
Source: Front Plant Sci. 2026 Jul 10;17:1822204. doi: 10.3389/fpls.2026.1822204 (PMC13395883; doi:10.3389/fpls.2026.1822204)
Supplement: Supplementary file 1 [file DataSheet1.pdf]

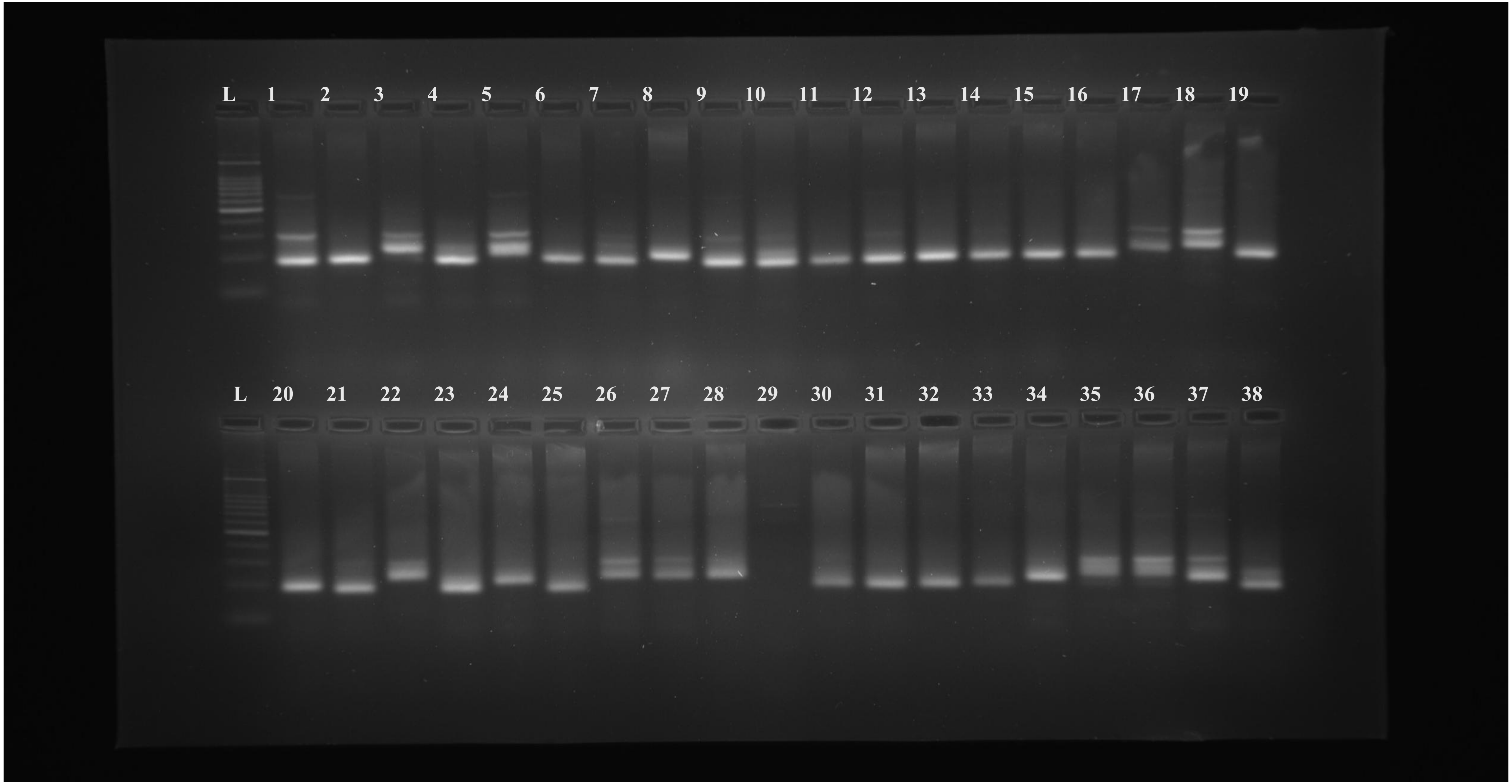

Supplementary Figure S5. Original uncropped gel image corresponding to Figure 5A showing amplification of the **RM247 marker**. Lane L represents the **100 bp DNA ladder**, and lanes 1–38 correspond to individual rice accessions analyzed in the study.

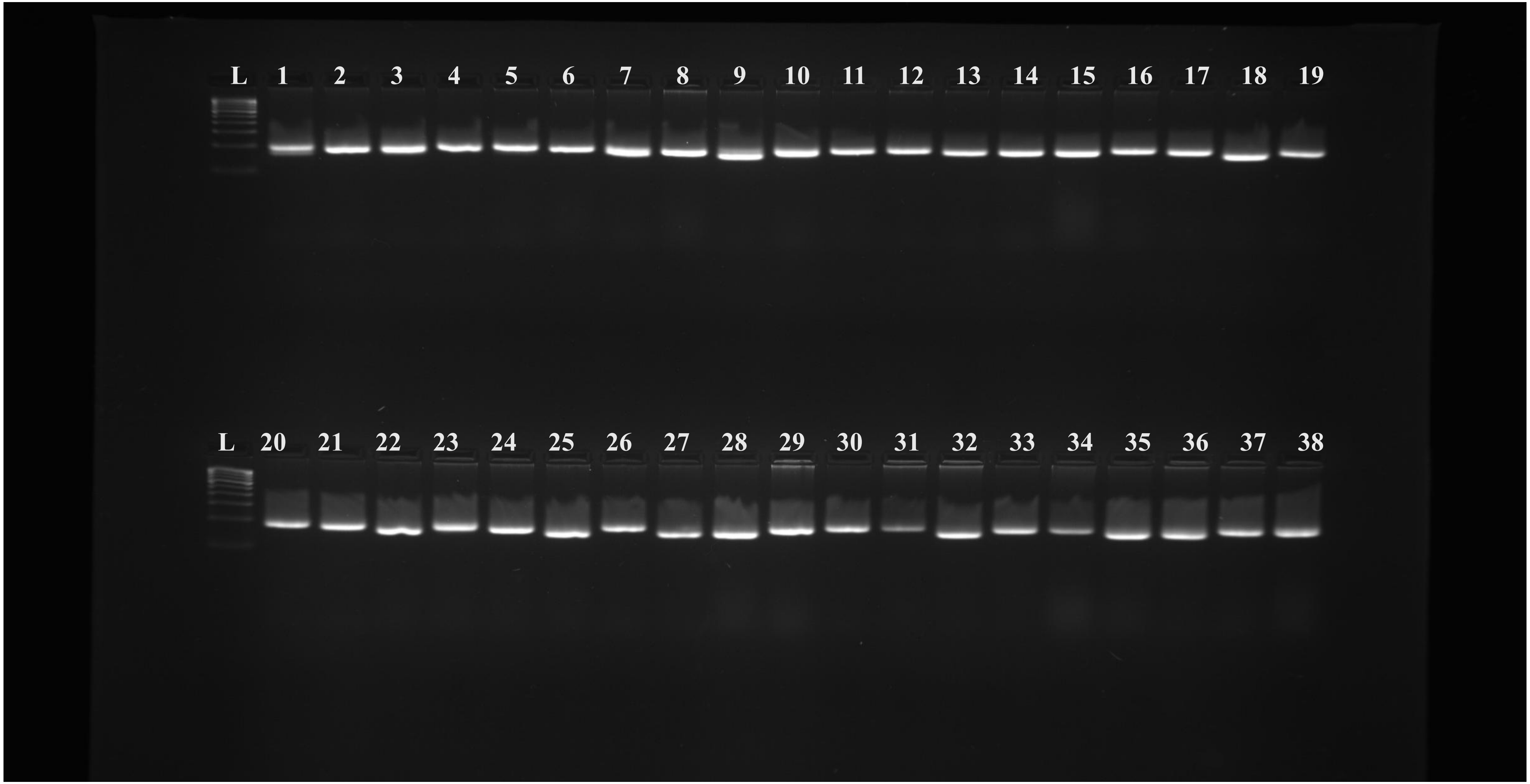

Supplementary Figure S6. Original uncropped gel image corresponding to Figure 5B showing amplification of the **RM231 marker**. Lane L represents the **100 bp DNA ladder**, and lanes 1–38 correspond to individual rice accessions analyzed in the study.

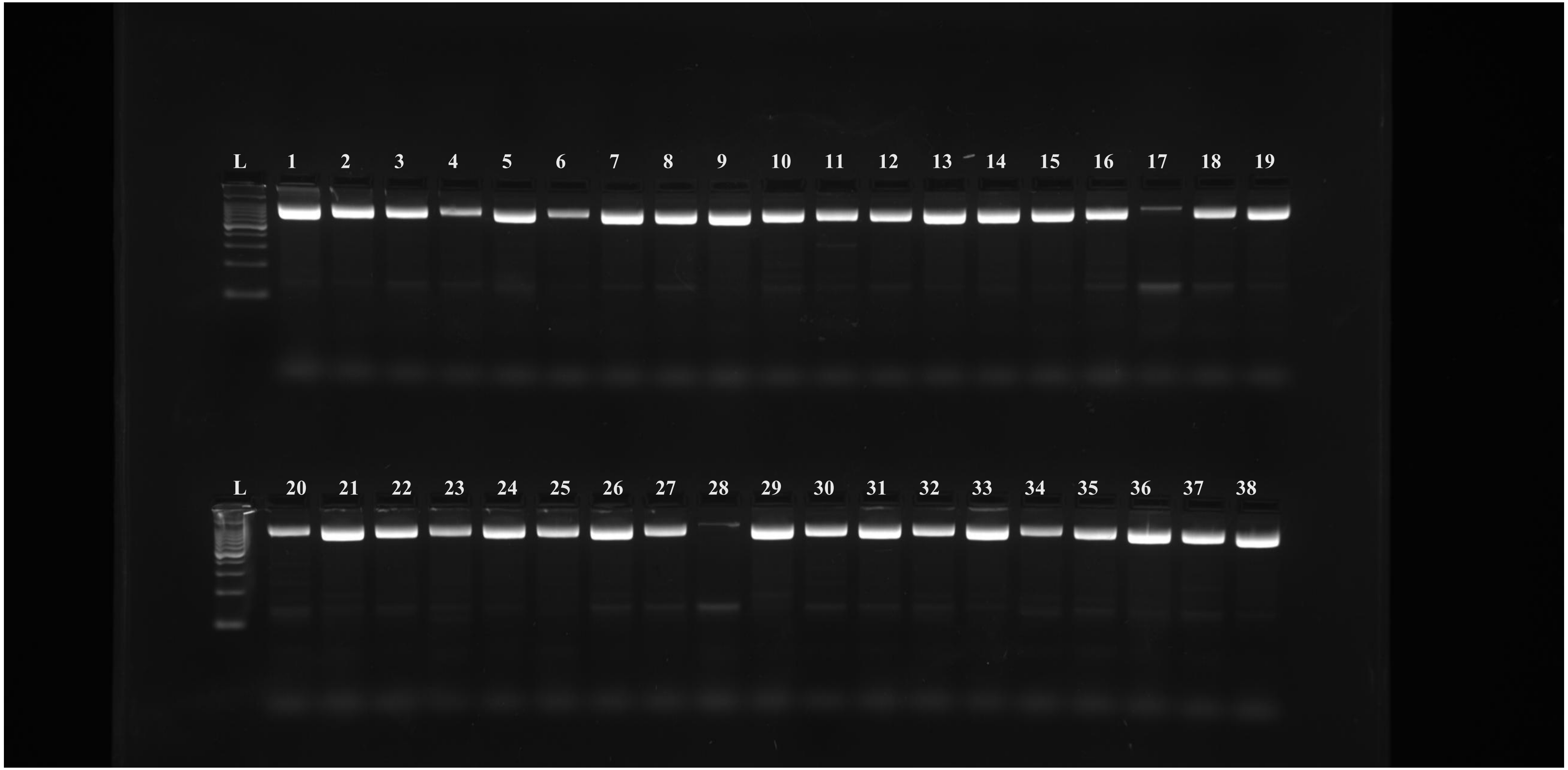

Supplementary Figure S7. Original uncropped gel image corresponding to Figure 5c showing amplification of the gene specific marker OsYSL2b. Lane L represents the 100 bp DNA ladder, and lanes 1–38 correspond to individual rice accessions analyzed in the study.
