# Supplementary material for: Elite donors and stable marker–trait associations for grain iron and zinc biofortification in rice under organic and inorganic production systems
Source: Front Plant Sci. 2026 Jul 10;17:1822204. doi: 10.3389/fpls.2026.1822204 (PMC13395883; doi:10.3389/fpls.2026.1822204)
Supplement: Supplementary file 2 [file DataSheet2.pdf]

Supplementary table S1. List of genotypes used in the study

S.No.	Germplasm	Category	Grain colour	Source
1	Adukkan	Landrace	C	TNAU, Coimbatore
2	Annai komban	Landrace	W	TNAU, Coimbatore
3	Annam alagi	Landrace	C	Farmers' Field
4	Arcot Kitchadi	Landrace	W	Farmers' Field
5	Basmati	Landrace	W	TNAU, Coimbatore
6	Bavani	Landrace	W	TNAU, Coimbatore
7	Buthakaima	Landrace	C	TNAU, Coimbatore
8	Chinna adukku nel	Landrace	C	TNAU, Coimbatore
9	Chinnar	Landrace	C	TNAU, Coimbatore
10	Chithiraikar	Landrace	C	AC&RI, Madurai
11	Edakkal	Landrace	C	TNAU, Coimbatore
12	Garudan samba	Landrace	W	TNAU, Coimbatore
13	Illupaipoo samba	Landrace	W	AC&RI, Madurai
14	Indhurani	Landrace	W	TNAU, Coimbatore
15	Jasmine	Landrace	W	Farmers' Field
16	Kadai kaluthan	Landrace	W	Farmers' Field
17	Kalluputhan	Landrace	C	AC&RI, Madurai
18	Kaluyundaiyan	Landrace	C	AC&RI, Madurai
19	Kamban samba	Landrace	W	TNAU, Coimbatore
20	Karunkuruvai	Landrace	C	TNAU, Coimbatore
21	Karuppu kavuni	Landrace	C	TNAU, Coimbatore
22	Karuthukkar	Landrace	C	Farmers' Field
23	Kattuyanam	Landrace	C	TNAU, Coimbatore
24	Kitchadi samba	Landrace	W	TNAU, Coimbatore
25	Kitchali samba	Landrace	W	Farmers' Field
26	Kochin samba	Landrace	W	TNAU, Coimbatore
27	Kuthiraivali samba	Landrace	W	Farmers' Field
28	Kuzhiyadichan	Landrace	C	AC&RI, Madurai
29	Mani samba	Landrace	W	TNAU, Coimbatore
30	Mappillai samba	Landrace	C	TNAU, Coimbatore
31	Milagu samba	Landrace	W	TNAU, Coimbatore
32	Nattu basmati	Landrace	W	Farmers' Field
33	Navara	Landrace	C	AC&RI, Madurai
34	Nei kitchadi	Landrace	W	Farmers' Field
35	Ottu kitchadi I	Landrace	W	TNAU, Coimbatore
36	Ottu kitchadi II	Landrace	W	Farmers' Field
37	Palkudai valzhai	Landrace	W	Farmers' Field
38	Pisini	Landrace	C	TNAU, Coimbatore
39	Polinel	Landrace	W	Farmers' Field
40	Ponmani samba	Landrace	W	TNAU, Coimbatore

41	Poongar	Landrace	C	AC&RI, Madurai
42	Ramakali	Landrace	C	TNAU, Coimbatore
43	Rathasali I	Landrace	C	TNAU, Coimbatore
44	Rathasali II	Landrace	C	Farmers' Field
45	Samba mosanam	Landrace	C	Farmers' Field
46	Seeraga samba	Landrace	W	TNAU, Coimbatore
47	Sempalai	Landrace	W	Farmers' Field
48	Sempuli samba	Landrace	W	TNAU, Coimbatore
49	Sengalapattu sirumani	Landrace	W	Farmers' Field
50	Sivan samba	Landrace	W	TNAU, Coimbatore
51	Sivappu kavuni	Landrace	C	TNAU, Coimbatore
52	Swarna malli	Landrace	W	TNAU, Coimbatore
53	Thanga samba	Landrace	W	Farmers' Field
54	Thooyamalli	Landrace	W	Farmers' Field
55	Thulasivasanai seeraga samba	Landrace	W	TNAU, Coimbatore
56	Vadakathi samba	Landrace	C	TNAU, Coimbatore
57	Vaigunda Red	Landrace	C	TNAU, Coimbatore
58	Vaigunda W	Landrace	W	TNAU, Coimbatore
59	Valan samba	Landrace	C	TNAU, Coimbatore
60	Varakkal	Landrace	C	TNAU, Coimbatore
61	Vasanai seeraga samba	Landrace	W	TNAU, Coimbatore
62	Vellai kudai valzhai	Landrace	C	TNAU, Coimbatore
63	ADT 46	Variety	W	RRS, Aduthurai
64	ADT 51	Variety	W	RRS, Aduthurai
65	ADT 53	Variety	W	RRS, Aduthurai
66	ADT 54	Variety	W	RRS, Aduthurai
67	Anna (R) 4	Variety	W	AC&RI, Madurai
68	ASD 16	Variety	W	AC&RI, Madurai
69	ADT 37	Variety	W	AC&RI, Madurai
70	CO 51	Variety	W	TNAU, Coimbatore
71	CO 54	Variety	W	TNAU, Coimbatore
72	CO 55	Variety	W	TNAU, Coimbatore
73	CO 56	Variety	W	TNAU, Coimbatore
74	CR 1009	Variety	W	AC&RI, Madurai
75	IR 50	Variety	W	AC&RI, Madurai
76	TRY 1	Variety	W	AC&RI, Trichy
77	TRY 5	Variety	W	AC&RI, Trichy
78	TKM 9	Variety	C	KVK, Tirur
79	TKM 11	Variety	W	KVK, Tirur
80	TKM 13	Variety	W	KVK, Tirur

Supplementary Table S2. Environmental and soil characteristics of experimental sites under organic and inorganic production systems (2023–2024)

	Organic site		Inorganic site	
	**2023**	**2024**	**2023**	**2024**
**Field details**				
Location	12°58'7" N - 79°9'40" E		12°57'54" N- 79°10'40" E	
Altitude	202 m			
**Weather conditions**				
Temperature (° C)	25 - 34	21 - 33	25 - 34	21 - 33
Relative humidity (%)	85	81	85	81
Rainfall (mm)	913	1443	913	1443
**Soil properties**				
pH	7.3	7.5	7.15	7.4
EC (mS/cm)	0.56	0.74	1.9	0.26
Organic carbon (%)	1.83	1.81	1.85	1.61
Available N (mg/Kg)	65.6	41.93	18.95	30.87
Available P (mg/Kg)	8.84	7.4	8.09	14.82
Available K (mg/Kg)	127	215	254	169
Available Zn (mg/Kg)	0.47	0.6	1.43	2.05
Available Fe (mg/Kg)	8.26	22.2	11.64	24.8

Supplementary Table S3. List of SSR markers used in the study

S. No.	Marker	Chromosome number	Annealing Temperature	Product Size (bp)	Forward primer (5'-3')	Reverse primer (5'-3')	Reference
1	RM3735	4	55	138	GCGACCGATCAGCTAGCTAG	ATAACTCCTCCCTTGCTGCC	(Anadhu et al., 2025)
2	RM 247	12	55	131	AAGGCGAACTGTCCTAGTGAAGC	CAGGATGTTCTTGCCAAGTTGC	(Anuradha et al., 2012)
3	RM 493	1	55	211	TAGCTCCAACAGGATCGACC	GTACGTAAACGCGGAAGGTG	
4	RM237	1	55	130	CAAATCCCGACTGCTGTCC	TGGGAAGAGAGCACTACAGC	
5	RM 541	6	55	158	TATAACCGACCTCAGTGCCC	CCTTACTCCCATGCCATGAG	
6	RM 248	7	55	102	TCCTTGTGAAATCTGGTCCC	GTAGCCTAGCATGGTGCATG	
7	RM 260	12	55	111	ACTCCACTATGACCCAGAG	GAACAATCCCTTCTACGATCG	
8	RM 152	8	55	151	GAAACCACCACACCTCACCG	CCGTAGACCTTCTTGAAGTAG	
9	RM 517	3	55	266	GGCTTACTGGCTTCGATTTG	CGTCTCCTTTGGTTAGTGCC	
10	RM 447	8	55	111	CCCTTGTGCTGTCTCCTCTC	ACGGGCTTCTTCTCCTTCTC	(Brar et al., 2014)
11	RM 205	9	55	122	CCTAAGAGGAGCCATCTAACAACTGG	CTTGGATATACTGGCCCTTCACG	
12	OsNAS2a	3	56	216-232	CGGTGTTCGACAACTACGAC	GATCACCCCGGCCTTCTC	(Namdev, 2020)
13	OsMTP1a	5	51	144-164	TGTGCTTTCGCTGTAACCAG	CCTGGCAGTCTTTTCCAACT	
14	OsNAS3f	7	51	176-190	CATTGCACTCCAATCCAAAA	TTCTCGATCACCTCCTCCTC	
15	RM 3644	8	55	173	GAAGAGAGTGGGAGGATGGG	AATTTGTGTGCTCCTCCACC	(Kiranmayi et al., 2014)
16	RM 259	1	55	162	TGGAGTTGAGAGGAGGGG	CTTGTTGCATGGTGCCATGT	(Lu et al., 2008)
17	RM 234	7	55	156	ACAGTATCCAAGGCCCTGG	CACGTGAGACAAAGACGGAG	
18	RM 2848	1	55	262	AATGTATTAGGATAAATGCGAAG	GAACGAACGTGAATGAGAAC	(Priyanka Walia, 2015)
19	RM228	10	55	154	TCTAACTCTGGCCATTAGTCCTTGG	AAGTAGACGAGGACGACGACAGG	
20	RM 72	8	55	166	CCGGCGATAAAACAATGAG	GCATCGGTCCTAACTAAGGG	
21	RM 339	8	55	148	GTAATCGATGCTGTGGGAAG	GAGTCATGTGATAGCCGATATG	(Pradhan et al., 2020)
22	RM 3412	1	55	211	AAAGCAGGTTTTCCTCCTCC	CCCATGTGCAATGTGTCTTC	(Raza et al., 2020)
23	RM 8094	1	55	209	AAGTTTGTACACATCGTATACA	CGCGACCAGTACTACTACTA	
24	RM 5607	2	56	107	AAAGCAGGTTTTCCTCCTCC	CCCATGTGCAATGTGTCTTC	
25	RM 335	4	55	104	GTACACACCCACATCGAGAAG	GCTCTATGCGAGTATCCATGG	
26	RM 430	5	55	173	AAACAACGACGTCCCTGATC	GTGCCTCCGTGGTTATGAAC	

27	RM 190	6	55	124	CTTTGTCTATCTCAAGACAC	TTGCAGATGTTCTTCCTGATG	
28	RM 110	2	55	156	TCGAAGCCATCCACCAACGAAG	TCCGTACGCCGACGAGGTCGAG	(Roja, 2011)
29	OSNRAMP7	12	55	1000	CGGGGCAGACTAGTACCATAACG	CAGCAAGAGATAGCCATTGATCG	
30	OsYSL2b	2	58	980	TCACTGCTAAGAGCCTGCAT	CTAGCTTCCGGGAGTGAACTT	(TaANNIDI, 2019)
31	OsZIP3b	4	58	370	CCTGCTGAGGCTGAGTTGAA	CGAGAACAAAGTAACAGGCTGC	
32	RM 243	1	55	116	GATCTGCAGACTGCAGTTGC	AGCTGCAACGATGTTGTCC	(Swamy et al., 2011)
33	RM 488	1	55	177	CAGCTAGGGTTTTGAGGCTG	TAGCAACAACCAGCGTATGC	
34	RM 231	3	55	182	CCAGATTATTTCCTGAGGTC	CACTTGCATAGTTCTGCATTG	
35	RM 223	8	55	165	GAGTGAGCTTGGGCTGAAAC	GAAGGCAAGTCTTGGCACTG	(Swamy et al., 2018)
36	RM 287	11	55	118	TTCCCTGTAAGAGAGAAATC	GTGTATTTGGTGAAAGCAAC	
37	RM 7364	9	50	204	TTTCGTGGATGGAGGGAGTACG	TGGCGACTTATGAGCGTTTGTAGG	

Supplementary Table S4. Agronomic performance of genotypes showing overlapping high grain Fe and Zn under organic and inorganic production systems

Organic Production System									
	**Fe**	**Zn**	**DFF**	**PH**	**PL**	**NPTP**	**HSW**	**NFGPP**	**SPY**
Annam alagi	19.82	33.05	87.93	119.94	20.17	20.63	2.22	90.67	24.37
Sempalai	18.07	31.28	87.93	119.94	20.17	20.63	2.22	90.67	24.37
Valan samba	17.72	29.77	95.99	130.51	25.13	23.22	2.63	108.61	42.35
Garudan samba	23.05	22.6	113.21	168.39	24.14	17.81	2.21	163.69	34.21
Sengalpattu sirumani	18.5	27.23	119.89	140.90	27.08	30.28	2.25	133.06	59.58
Vellai kudai valzhai	17.62	25.29	91.44	170.94	27.81	26.23	2.13	142.92	48.82
Mani samba	17.44	28.58	99.90	148.61	26.60	23.70	2.36	142.30	46.41
Illupaipoo samba	16.89	24.41	92.97	141.58	22.06	22.50	1.53	99.41	39.29
Sivappu kavuni	16.18	23.62	100.31	160.06	26.19	26.17	2.40	164.57	46.86
Basmati	13.71	38.38	97.92	126.39	24.60	23.12	2.58	130.56	36.30
Arcot kitchadi	15.32	31.6	103.83	154.39	25.17	23.83	2.13	158.03	41.08
Rathasali II	15.71	31.28	95.04	111.34	18.97	37.02	1.56	85.02	39.81
Vaigunda Red	15.52	30.74	94.34	147.72	23.40	24.17	2.46	138.04	40.88
Mappillai samba	15.13	30.16	119.40	175.34	22.38	17.19	3.04	111.23	53.68
**Inorganic Production System**									
	**Fe**	**Zn**	**DFF**	**PH**	**PL**	**NPTP**	**HSW**	**NFGPP**	**SPY**
Annam alagi	17.09	29.22	85.72	100.33	20.06	19.18	2.22	81.89	28.01
Sempalai	15.79	28.21	89.65	136.18	25.34	23.19	2.30	141.77	39.85
Valan samba	17.55	29.39	88.83	141.40	28.60	25.01	2.79	120.03	41.70
Garudan samba	19.64	24.59	113.57	144.81	24.71	20.35	2.16	157.20	35.48
Sengalpattu sirumani	15.98	24.46	114.66	132.39	28.09	20.16	2.04	148.72	39.39
Vellai kudai valzhai	15.85	25.28	88.72	153.23	24.83	24.45	2.21	161.19	39.76
Mani samba	18.54	27.70	96.22	132.05	24.83	35.06	2.39	161.78	59.78
Illupaipoo samba	18.01	26.06	90.78	127.22	23.99	19.27	1.55	93.78	27.37
Sivappu kavuni	16.53	23.68	97.84	154.90	24.98	23.57	2.38	157.66	40.88
Basmati	12.71	30.84	93.87	113.07	25.42	19.16	2.62	135.72	43.57
Arcot kitchadi	14.54	27.86	105.26	162.04	24.45	22.52	2.70	160.78	41.34
Rathasali II	14.30	28.1	91.10	128.05	22.48	29.96	1.31	92.83	41.08
Vaigunda Red	15.57	29.13	94.06	142.83	22.68	25.38	2.59	176.30	39.51
Mappillai samba	11.69	28.4	115.49	164.21	26.10	22.82	3.14	143.07	43.44
